# CRISPR/Cas9-mediated CysLT1R deletion reverses synaptic failure, amyloidosis and cognitive impairment in APP/PS1 mice

**DOI:** 10.18632/aging.202501

**Published:** 2021-02-11

**Authors:** Fang Chen, Shunchang Fang, Yifeng Du, Arijit Ghosh, Miranda N. Reed, Yan Long, Vishnu Suppiramaniam, Susu Tang, Hao Hong

**Affiliations:** 1 Department of Pharmacology, China Pharmaceutical University, Nanjing, China; 2 Department of Pharmacy, The First Affiliated Hospital of Xiamen University, Xiamen, China; 3 Department of Drug Discovery and Development, School of Pharmacy, Auburn University, Auburn, AL 36849, USA

**Keywords:** Alzheimer’s disease, cysteinyl leukotrienes receptor 1, cognition, amyloidogenesis, synaptic plasticity

## Abstract

As a major pathological hallmark of Alzheimer’s disease (AD), amyloid-β (Aβ) is regarded as a causative factor for cognitive impairment. Extensive studies have found Aβ induces a series of pathophysiological responses, finally leading to memory loss in AD. Our previous results demonstrated that cysteinyl leukotrienes receptor 1 (CysLT_1_R) antagonists improved exogenous Aβ-induced memory impairment. But the role of CysLT_1_R in AD and its underlying mechanisms still remain elusive. In this study, we investigated CysLT_1_R levels in AD patients and APP/PS1 mice. We also generated APP/PS1-CysLT_1_R^-/-^ mice by clustered regulatory interspaced short palindromic repeats (CRISPR)/CRISPR-associated protein 9 (Cas9)-mediated CysLT_1_R deletion in APP/PS1 mice and studied the effect of CysLT_1_R knockout on amyloidogenesis, synapse structure and plasticity, cognition, neuroinflammation, and kynurenine pathway. These attributes were also studied after lentivirus-mediated knockdown of CysLT_1_R gene in APP/PS1 mice. We found that CysLT_1_R knockout or knockdown could conserve synaptic structure and plasticity, and improve cognition in APP/PS1 mice. These effects were associated with concurrent decreases in amyloid processing, reduced neuroinflammation and suppression of the kynurenine pathway. Our study demonstrates that CysLT_1_R deficiency can mediate several beneficial effects against AD pathogenesis, and genetic/pharmacological ablation of this protein could be a potential therapeutic option for AD.

## INTRODUCTION

Amyloid-β (Aβ) is the main pathological hallmark of Alzheimer’s disease (AD), and neurotoxic effects induced by Aβ have been well documented to be a causative factor of the disease. AD is also characterized by neurofibrillary tangles made up of hyperphosphorylated tau, and elevated neuroinflammation in the brain, which induce impairment of hippocampal synaptic plasticity and may lead to neuronal apoptosis, finally leading to cognitive decline in the affected persons [1]. Neuroinflammation has become an increasingly stressed topic in AD pathology which is characterized by the activation of astrocytes and microglia that trigger a vicious cycle of inflammatory responses through the increased release of pro-inflammatory cytokines [2]. These inflammatory cytokines have been reported to augmenting Aβ formation and resulting in neuronal damage [3]. Importantly, treatments blocking the inflammatory responses can reverse Aβ-induced neuroinflammation and memory impairment [4]. Therefore, therapeutic targeting of neuroinflammation in AD is becoming the focus for future studies.

Cysteinyl leukotrienes (Cys-LTs) are a group of the inflammatory lipid molecules that include LTC4, LTD4, and LTE4, and are generated by the activation of 5-lipoxygenase (5-LOX) pathway of the arachidonic acid metabolism [5]. Cys-LTs initiate inflammatory signaling cascades by two major G-protein coupled receptors: CysLT_1_R and CysLT_2_R, the roles of which have traditionally been examined in relation to asthma and other allergic diseases [6]. However, in the recent years, increasing evidence has backed their possible involvement in the pathophysiology of AD, Parkinson’s disease (PD), and other neurological disorders [7, 8]. We previously have found the involvement of CysLT_1_R in Aβ-induced neurotoxicity, as the neuroinflammation and memory impairment induced by Aβ_1-42_ are ameliorated through administration of selective CysLT_1_R antagonists such as montelukast and pranlukast [9]. Moreover, we have suggested that CysLT_1_R is strongly related with LPS-induced neuroinflammation, apoptosis and memory impairment, and through pretreating mice with pranlukast [10]. However, the precise mechanisms by which CysLT_1_R mediates AD-related learning and memory impairment were unknown.

Many studies have indicated that elevated Aβ_1-42_ is correlated with proinflammatory cytokines induced-kynurenine pathway (KP) activation [11]. KP is the main metabolic pathway of tryptophan degradation producing a series of metabolites [12]. Quinolinic acid (QUIN), one metabolite from KP, is considered to be an endogenous N-methyl-D-aspartate receptor (NMDAR) agonist with strong excitotoxic properties, which leads to glutamatergic excitotoxicity through overactivation of NMDARs in AD [13]. In additions, QUIN is present in neurons, microglia and astrocytes around senile plaques and neurofibrillary tangles in the hippocampus of AD patients [14]. As a rate-limit enzyme involved in the main pathway towards NAD^+^ synthesis, kynureninase (KYNU) promotes the production of QUIN in KP and is also involved in AD pathogenesis. Since CysLT_1_R mediates Aβ-induced neurotoxicity following by elevated proinflammatory cytokines, which in turn induces kynurenine pathway dysregulation, we hypothesized that CysLT_1_R could be important for KP regulation, leading to neuroinflammation and related cognitive impairment. To test this hypothesis and specifically elucidate the role of CysLT_1_R during AD pathogenesis, we downregulated the expression levels of CysLT_1_R by knockout or knockdown of the CysLT_1_R gene in APP/PS1 mice for the first time, and examined the effects of CysLT_1_R manipulation on amyloidosis, synaptic plasticity, cognition, neuroinflammation, and KP regulation.

## RESULTS

### CysLT_1_R expression is upregulated in APP/PS1 mice and AD patients

**Figure 1:**
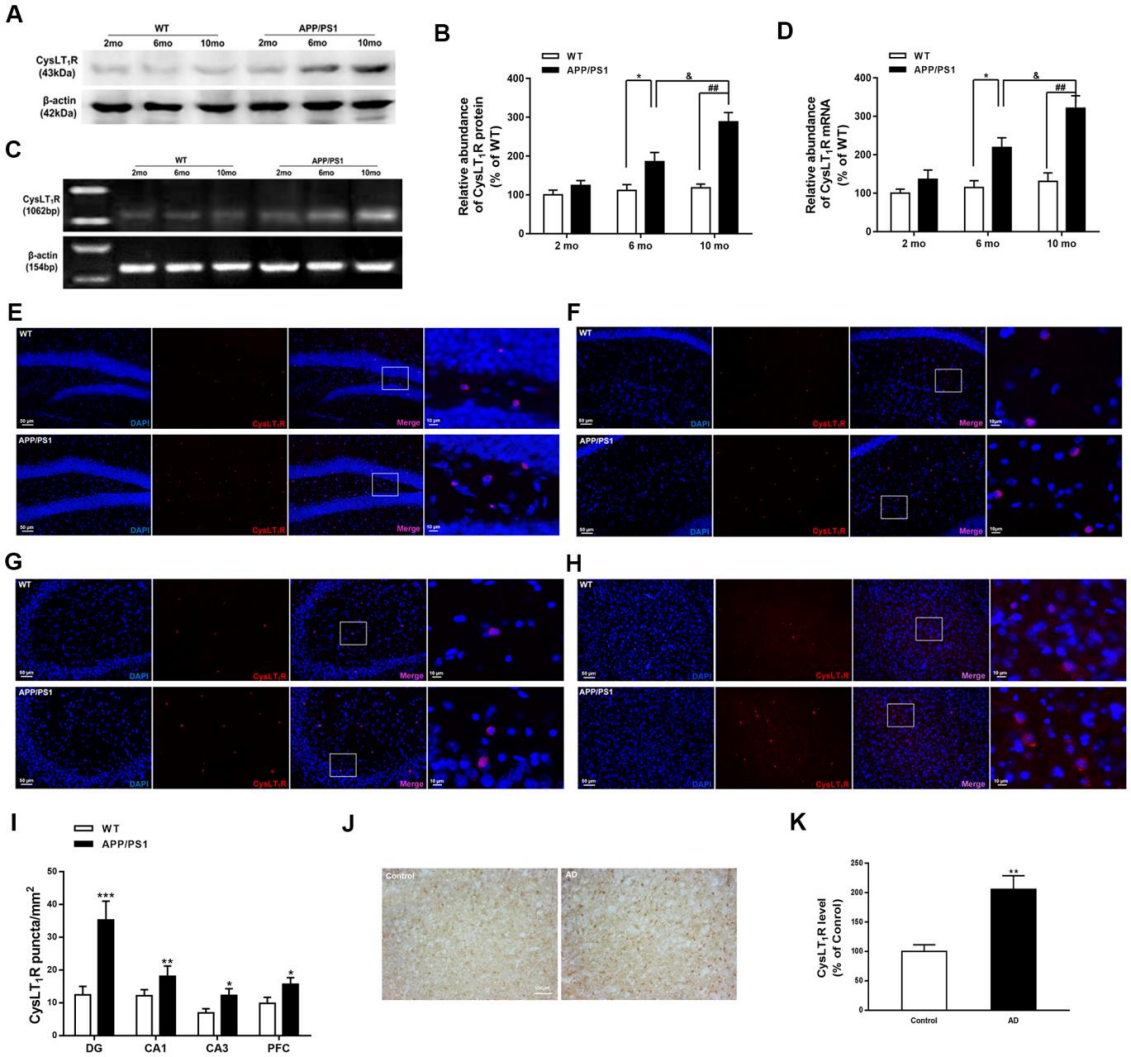
**CysLT_1_R expression is upregulated in APP/PS1 mice and AD patients.** (**A**) Representative immunoblots of CysLT_1_R protein in the hippocampus of APP/PS1 mice and WT mice at the age of 2, 6 and 10 months. (**B**) Quantification of CysLT_1_R protein levels was expressed as the ratio (in %) of WT group. Data are expressed as mean ± SEM, n = 4, *P < 0.05 vs. 6-month-old WT mice; ^##^*P* < 0.01 vs. 10-month-old WT mice; ^&^*P* < 0.05 vs. 6-month-old APP/PS1 mice. (**C**) RT-PCR detection of CysLT_1_R mRNA in the hippocampus of APP/PS1 mice and WT mice at the age of 2, 6 and 10 months. (**D**) Quantification of CysLT_1_R mRNA levels was expressed as the ratio (in %) of WT group. Data are expressed as mean ± SEM, n = 4, *P < 0.05 vs. 6-month-old WT mice; ^##^*P* < 0.01 vs. 10-month-old WT mice; ^&^*P* < 0.05 vs. 6-month-old APP/PS1 mice. Immunofluorescence images of CysLT_1_R expression in the hippocampal DG (**E**), CA1 (**F**), CA3 (**G**), and prefrontal cortex (**H**) in APP/PS1 mice and WT mice. Scale bar = 50 μm. (**I**) Quantification of CysLT_1_R in the brain sections of mice. Data are expressed as mean ± SEM, n = 4, *P < 0.05, **P < 0.01, ***P < 0.01 vs. WT mice. (**J**) CysLT_1_R levels in the brain sections from post-mortem AD patients and normal controls by immunohistochemical analyses. Scale bar = 100 μm. (**K**) Quantification of CysLT_1_R in the sections of human postmortem brains. Data are expressed as mean ± SEM, n = 4, **P<0.01 vs. control.

We first measured CysLT_1_R levels in APP/PS1 mice hippocampus. Both protein and mRNA levels of CysLT_1_R were elevated to about 2-fold and 3-fold in the hippocampus of 6-month-old (for protein, F [1, 6] = 2.473, *P<0.05*; for mRNA, F [1, 6] = 1.96, *P*<0.05) and 10-month-old APP/PS1 mice (for protein, F [1, 6] = 12.71, for mRNA, *P*<0.01; F [1, 6] = 2.213, *P*<0.01) compared to these in WT mice, respectively (Figure 1A–1D).

We next confirmed the elevated CysLT_1_R levels in brain sections from 10-month old APP/PS1 mice by immunofluorescence staining. APP/PS1 mice exhibited significant upregulation of CysLT_1_R in the dentate gyrus (DG; F [1, 10] = 4.926, *P*<0.001, Figure 1E–1I), CA1 (F [1, 10] = 1.978, *P*<0.01, Figure 1F–1I), and CA3 (F [1, 10] = 2.617, *P*<0.05, Figure 1G–1I) subregions of the hippocampus, as well as in the prefrontal cortex (PFC; F [1, 10] = 1.186, *P*<0.05, Figure 1H, 1I). Moreover, expressions of CysLT_1_R in neurons, astrocytes, and microglia in the DG of the APP/PS1 mice were significantly elevated (for astrocytes, F [1, 10] = 2.901, *P*<0.001, Supplementary Figure 1A–1D; for neurons, F [1, 10] = 4.874, *P*<0.01, Supplementary Figure 1B–1D; for microglia, F [1, 10] = 2.708, *P*<0.05, Supplementary Figure 1C, 1D).

We further confirmed elevated protein levels of CysLT_1_R in AD patients by immunohistochemical analyses. A 2-fold elevation in CysLT_1_R levels (F [1, 14] = 4.491, *P*<0.01, Figure 1J, 1K) in the prefrontal cortex of AD patients was observed compared to age-matched controls. The detail information of AD patients and controls has been shown in the Supplementary Table 1. Altogether, CysLT_1_R levels are upregulated in both APP/PS1 mice and AD patients compared to controls. Moreover, CysLT_1_R protein and mRNA levels in APP/PS1 mice are increased with age.

### CysLT_1_R deletion enhances hippocampal synaptic plasticity in APP/PS1 mice

**Figure 2:**
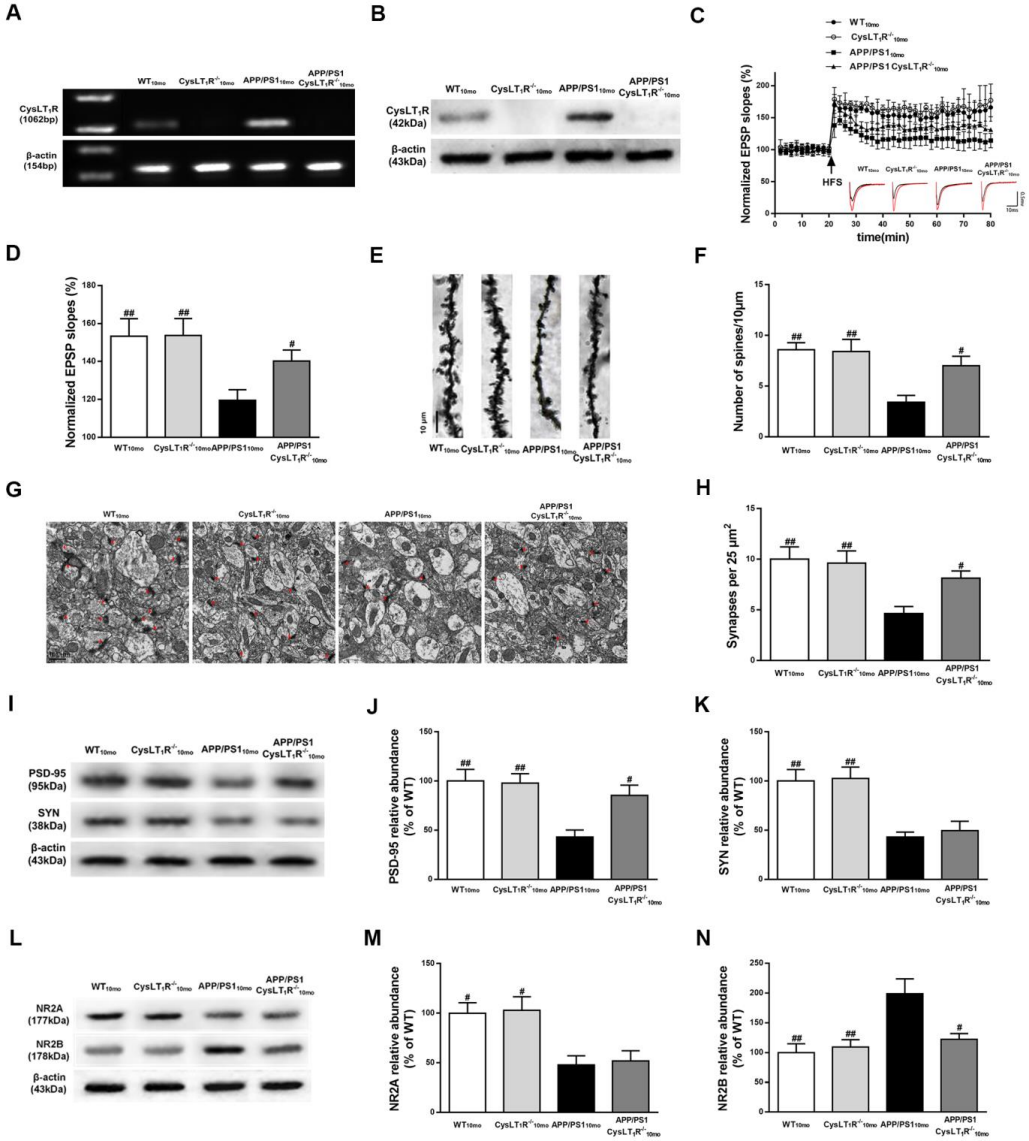
**CysLT_1_R deficiency enhances hippocampal synaptic plasticity in APP/PS1 mice.** (**A**) Western blot detection of CysLT_1_R protein in mice hippocampus. (**B**) RT-PCR assay of CysLT_1_R mRNA in mice hippocampus. (**C**) The induction of hippocampal LTP was assessed after high-frequency stimulation (HFS; indicated as an arrow) and recorded for 60 min post-induction. (**D**) Summary bar-graphs showing differences in mean values of fEPSPs slope during 55-60 min following the induction of LTP among genotypes. (**E**) Representative images of Golgi-impregnated dendrites in the hippocampus. Scale bar = 10 μm. (**F**) Statistical analysis of the average number of dendritic spines. (**G**) The synaptic density in the hippocampus was determined by electron microscopy. Scale bar = 1 μm. (**H**) Statistical analysis of synaptic density calculated as the number of synapses per 25 μm^2^. (**I**) Representative immunoblots of PSD-95 and SYN in mice hippocampus. Quantifications of (**J**) PSD-95 and (**K**) SYN protein levels were expressed as the ratio (in %) of WT group. (**L**) Representative immunoblots of NR2A and NR2B in mice hippocampus. Quantifications of (**M**) NR2A and (**N**) NR2B were expressed as the ratio (in %) of WT group. All values are expressed as mean ± SEM, n = 4-6, ^#^*P*<0.05, ^##^*P*<0.01, ^###^*P*<0.001 vs. APP/PS1 mice.

To determine whether CysLT_1_R deletion could improve hippocampal synaptic plasticity in APP/PS1 mice, we generated CysLT_1_R knockout mice (CysLT_1_R^-/-^) and bred them with APP/PS1 mice to create CysLT_1_R knockout APP/PS1 mice (APP/PS1-CysLT_1_R^-/-^). Identifications for CysLT_1_R^-/-^ mice and APP/PS1-CysLT_1_R^-/-^ mice have been provided in Supplementary Material II and III, respectively. Moreover, the deletion of CysLT_1_R have been further confirmed (Figure 2A, 2B). Electrophysiological analysis demonstrated that CysLT_1_R deletion in APP/PS1 mice could ameliorate the LTP deficits at Schaffer collateral pathways and CysLT_1_R deficiency didn’t affect LTP induction in WT mice (F [3, 12] = 8.743, *P* < 0.05, Figure 2C, 2D). The improvement in LTP was associated with a concurrent restoration in dendritic spine density in APP/PS1- CysLT_1_R^-/-^ mice (F [3, 16] = 7.069, *P* < 0.05, Figure 2E, 2F). The number of synapses was also evaluated by the presence of synaptic vesicles observed using an electron microscope. CysLT_1_R deletion significantly ameliorated synaptic losses in APP/PS1 mice (F [3, 28] = 6.096, *P* < 0.05, Figure 2G, 2H). We next analyzed the levels of the presynaptic and postsynaptic markers, synaptophysin (SYN) and PSD-95, respectively, in the mice hippocampus. Though both were reduced in APP/PS1 mice, CysLT_1_R deletion only restored the levels of PSD-95, but not of SYN, in APP/PS1-CysLT_1_R^-/-^ mice (for PSD-95, F [3, 12] = 7.187, *P* < 0.05, Figure 2I, 2J; and for SYN, F [3, 12] = 10.54, *P* > 0.05, Figure 2I–2K).

As NMDARs are crucial for mediating synaptic transmission and plasticity in mature excitatory glutamatergic synapses, as well as in excitotoxicity [15], we next examined the expression of NMDAR subunits, NR2A and NR2B. CysLT_1_R deficiency markedly decreased NR2B expression, but it had no effect on NR2A expression (for NR2A, F [3, 12] = 7.339, *P* > 0.05, Figure 2L, 2M; and for NR2B, F [3, 12] = 7.392, *P* < 0.05, Figure 2L–2N). Together, these results indicate that CysLT_1_R deletion ameliorates deficits in synaptic integrity and plasticity in APP/PS1 mice at 10 months of age.

### CysLT_1_R deletion inhibits amyloidogenesis in the hippocampus of APP/PS1 mice

**Figure 3:**
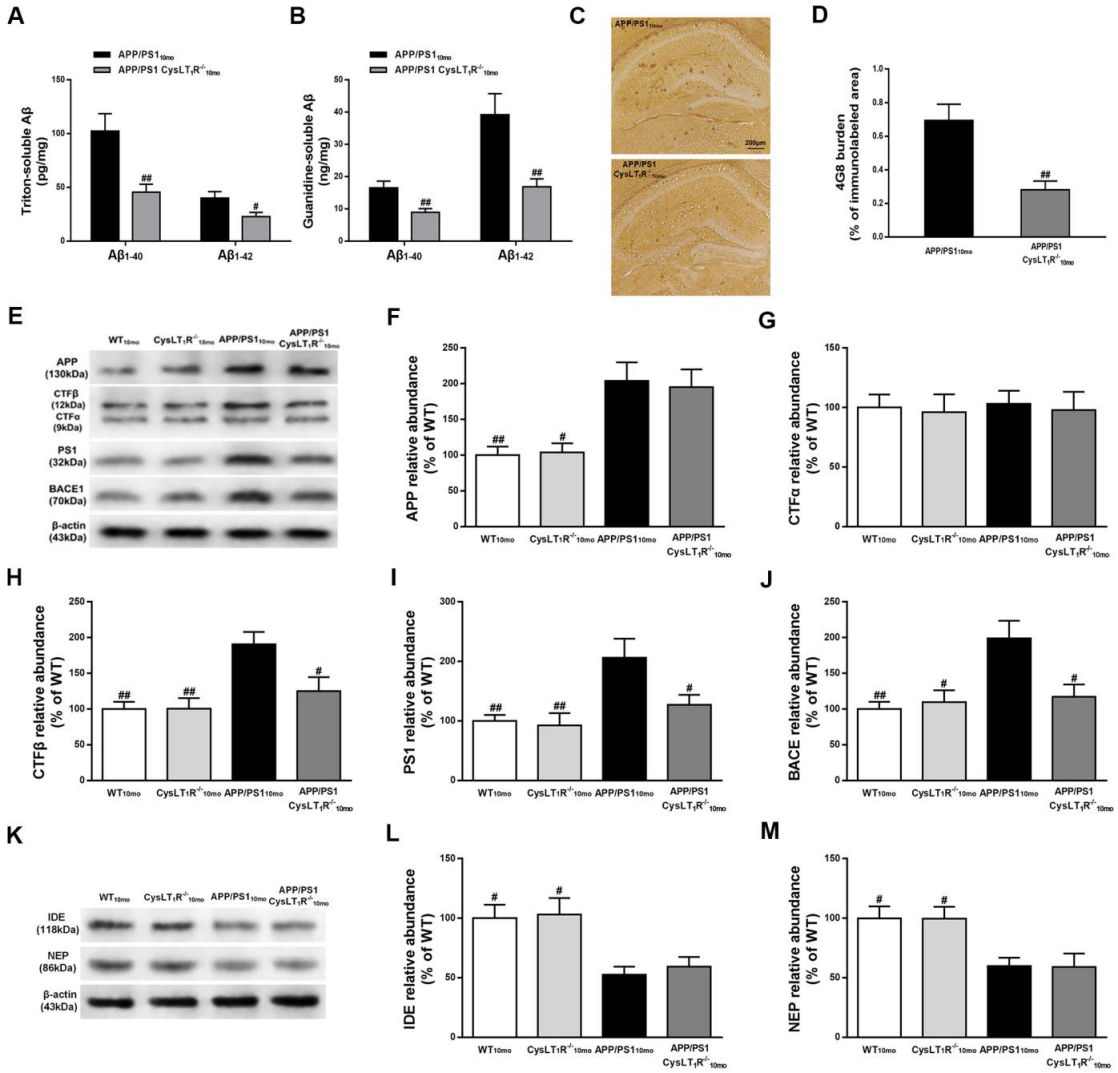
**CysLT_1_R deficiency inhibits amyloidogenesis in APP/PS1 mice hippocampus.** (**A**)The triton-soluble fractions and (**B**) the guanidine-soluble fractions of Aβ_1-40_ and Aβ_1-42_ in mice hippocampus were assessed by ELISA. (**C**) Aβ immunostaining with 4G8 antibody in hippocampus of mice. Scale bar = 200 μm. (**D**) The percentage of area covered by Aβ deposition was quantified. (**E**) Representative immunoblots of APP, CTFα, CTFβ, PS1 and BACE in the hippocampus of mice. Quantifications of (**F**) APP, (**G**) CTFα, (**H**) CTFβ, (**I**) PS1 and (**J**) BACE were expressed as the ratio (in %) of WT group. (**K**) Representative immunoblots of IDE and NEP in the hippocampus of mice. Quantifications of (**L**) IDE and (**M**) NEP were expressed as the ratio (in %) of WT group. All values are expressed as mean ± SEM, n = 4-6, ^#^*P*<0.05, ^##^*P*<0.01, ^###^*P*<0.001 vs. APP/PS1 mice.

To determine whether CysLT_1_R deletion reduces amyloid pathology, we measured triton-soluble and guanidine-soluble Aβ in APP/PS1-CysLT_1_R^-/-^ mice hippocampus. Aβ_1-40_ and Aβ_1-42_ in both triton-soluble fraction and guanidine-HCl extraction from APP/PS1-CysLT_1_R^-/-^ mice hippocampus were substantially reduced (triton-soluble: for Aβ_1-40_, F [1, 10] = 4.72, *P*<0.01, and for Aβ_1-42_, F [1, 10] = 2.382, *P*<0.05, Figure 3A; guanidine-soluble: for Aβ_1-40_, F [1, 10] = 3.258, *P*<0.01, and for Aβ_1-42_, F [1, 10] = 7.084, *P*<0.01, Figure 3B). Aβ deposition was also examined by immunostaining with 4G8 antibody on the brain sections of APP/PS1-CysLT_1_R^-/-^ mice. Compared to APP/PS1 mice (0.69 ± 0.10 %), Aβ deposition significantly reduced in the APP/PS1-CysLT_1_R^-/-^ mice hippocampus (0.28 ± 0.05 %) (F [1, 22] = 3.414, *P*<0.01, Figure 3C, 3D).

To further clarify the role of CysLT_1_R in Aβ production and degradation, we studied whether CysLT_1_R deletion affected amyloid precursor protein (APP) processing and Aβ degradation. While CysLT_1_R deletion did not alter hippocampal APP (F [3, 12]=7.922, *P*>0.05, Figure 3E, 3F) or CTFα expression (F [3, 12] = 0.052, *P* > 0.05, Figure 3E–3G), levels of CTFβ (F [3, 12] = 7.341, *P* < 0.01, Figure 3E–3H), PS1 (F [3, 12] = 6.098, *P* < 0.01, Figure 3E–3I), and BACE (F [3, 12] = 6.448, *P* < 0.01, Figure 3E–3J) were significantly reduced in APP/PS1-CysLT_1_R^-/-^ mice. Moreover, CysLT_1_R deletion did not restore IDE and NEP expression (for IDE, F [3, 12] = 6.573, *P* > 0.05; and for NEP, F [3, 12] = 5.861, *P* > 0.05, Figure 3K–3M). These results indicate that CysLT_1_R deletion inhibits amyloidogenesis in APP/PS1 mice hippocampus most likely via a decrease in amyloidogenic APP processing rather than an increase in Aβ degradation.

### CysLT_1_R knockout ameliorates cognitive decline in APP/PS1 mice

**Figure 4:**
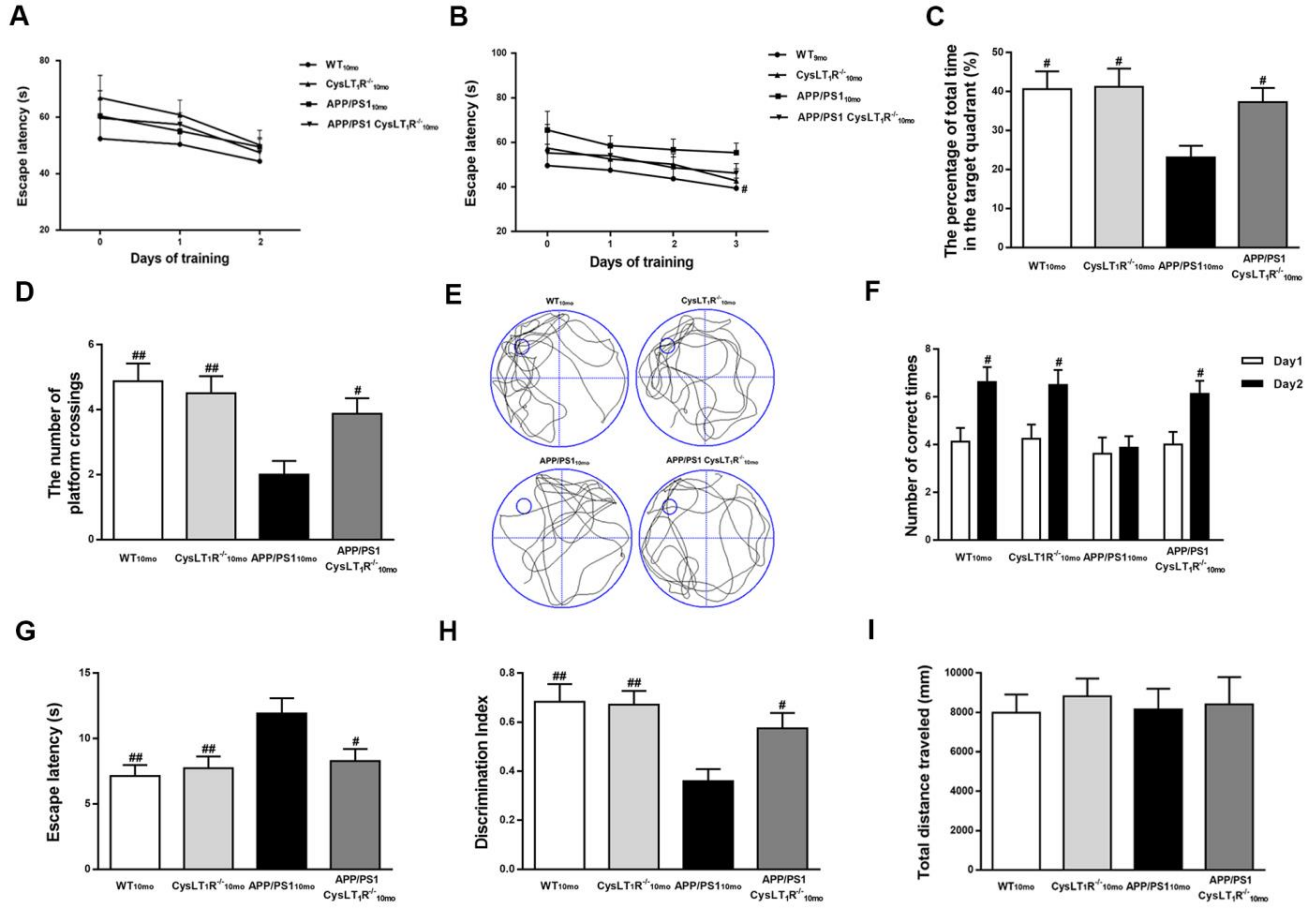
**CysLT_1_R deficiency ameliorates cognitive decline in APP/PS1 mice.** (**A**) The mean escape latency to the visible platform during day 1–2. (**B**) The mean escape latency to the hidden platform during day 3–5. (**C**) The percentage of time stayed in the target quadrant, and (**D**) numbers of platform crossings during day 6. (**E**) Representative swim paths of mice. In the Y-maze test, (**F**) the number of correct choices on days 1-2 and (**G**) the latency to enter the safe compartment on day 2. In NORT, (**H**) discrimination index shown by the time spent exploring the novel object relative to the total time spent exploring both novel and familiar objects. In open field test, (**I**) the total distance traveled was analyzed. All values are expressed as mean ± SEM, n = 8, ^#^*P*<0.05, ^##^*P*<0.01, ^###^*P*<0.001 vs. APP/PS1 mice.

To evaluate the effects of CysLT_1_R deficiency on spatial learning and memory, mouse behavior was tested by the MWM task. During day 1-2 (visible platform training), all groups exhibited the similar latencies, suggesting similar visual and motor functions among all the groups (effect of day, F [3, 284] = 4.563, *P* < 0.05; effect of group, F [3, 284] = 39.05, *P* > 0.05; effect of group-by-day interaction, F [3, 284] = 2.648, *P* > 0.05, Figure 4A). During day 3-5 (hidden platform training), APP/PS1-CysLT_1_R^-/-^ mice showed significant decreases in escape latency compared to APP/PS1 mice (effect of day, F [3, 412] = 9.638, *P* < 0.05; effect of group, F [3, 412] = 23.628, *P* < 0.05; effect of group-by-day interaction, F [3, 412] = 1.782, *P* > 0.05, Figure 4B). In the probe trial, CysLT_1_R deletion significantly increases the percentage of time stayed in the target quadrant (F [3, 28] = 4.405, *P* < 0.05, Figure 4C–4E) and the numbers of platform crossings in APP/PS1 mice (F [3, 28] = 6.55, *P* < 0.05, Figure 4D, 4E). Y-maze task evaluation also showed that impairment of working memory was alleviated (for the number of correct choices, F [3, 28] = 5.751, *P* < 0.05, Figure 4F; and for the escape latency, F [3, 28] = 5.053, *P* < 0.05, Figure 4G) by CysLT_1_R deficiency. Moreover, this was further confirmed by novel object recognition test (F [3, 28] = 6.045, *P* < 0.05, Figure 4H). Importantly, all groups traveled similar total distance in the open field test (F [3, 28] = 0.119, *P* > 0.05, Figure 4I), suggesting similar exploration capability and motor functioning.

### CysLT_1_R knockout alleviates neuroinflammation in the hippocampus of APP/PS1 mice

**Figure 5:**
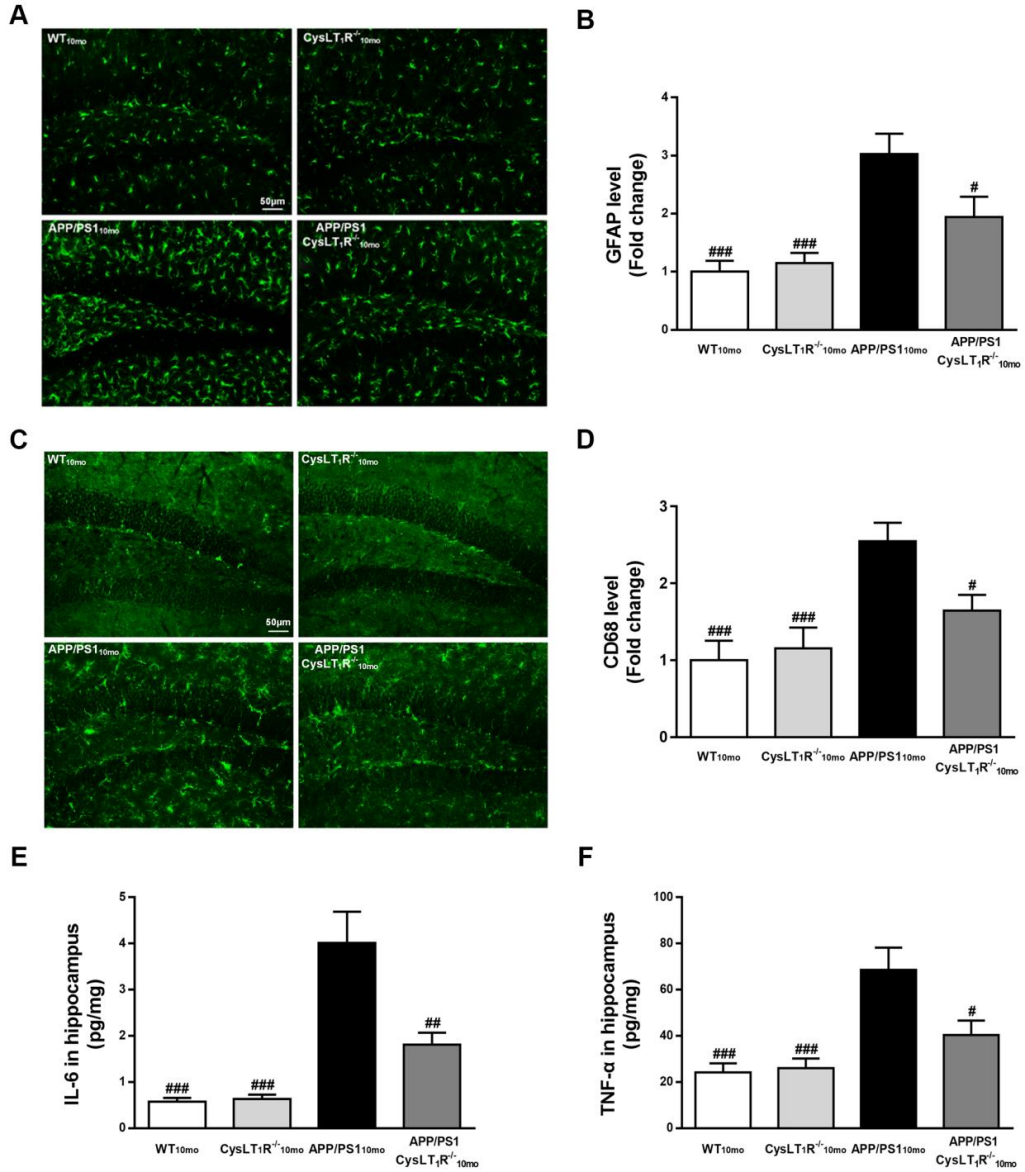
**CysLT_1_R deficiency alleviates neuroinflammation in APP/PS1 mice hippocampus.** (**A**) GFAP^+^ astrocytes in hippocampal sections from different groups were detected. Scale bar = 50 μm. (**B**) The percentage of GFAP^+^-area was quantified. (**C**) CD68^+^ microglia in hippocampal sections from different groups were detected. Scale bar = 50 μm. (**D**) The percentage of CD68^+^-area was quantified. (**E**) IL-6 and (**F**) TNF-α in mice hippocampus were assessed by ELISA. All values are expressed as mean ± SEM, n = 4-6, ^#^*P<0.05*, ^##^*P<0.01*, ^###^*P*<0.001 vs. APP/PS1 mice.

Our previous studies found that CysLT_1_R activated the NF-κB signaling pathway, leading to increased release of inflammatory cytokines [16]. To supplement our previous findings, we compared markers for activation of astrocytes and microglia using antibodies to glial fibrillary acidic protein (GFAP) and CD68, respectively. We observed that APP/PS1-CysLT_1_R^-/-^ mice showed the less GFAP^+^ astrocytes (F [3,44] = 11.11, *P* < 0.05, Figure 5A, 5B) and CD68^+^ microglia (F [3,44] = 8.098, *P* < 0.05, Figure 5C, 5D) in the DG. CysLT_1_R deletion also decreased the levels of proinflammatory cytokines including interleukin-6 (IL-6) and tumor necrosis factor-α (TNF-α) in APP/PS1 mice hippocampus (for IL-6, F [3, 20] = 19.29, *P* < 0.01, Figure 5E; and for TNF-α, F [3, 20] = 10.06, *P* < 0.05, Figure 5F). These data provide that CysLT_1_R deletion inhibits neuroinflammation, which may help ameliorating synaptic dysfunction and cognitive impairment.

### Kynurenine pathway is involved in CysLT_1_R-mediated synaptic dysfunction

**Figure 6:**
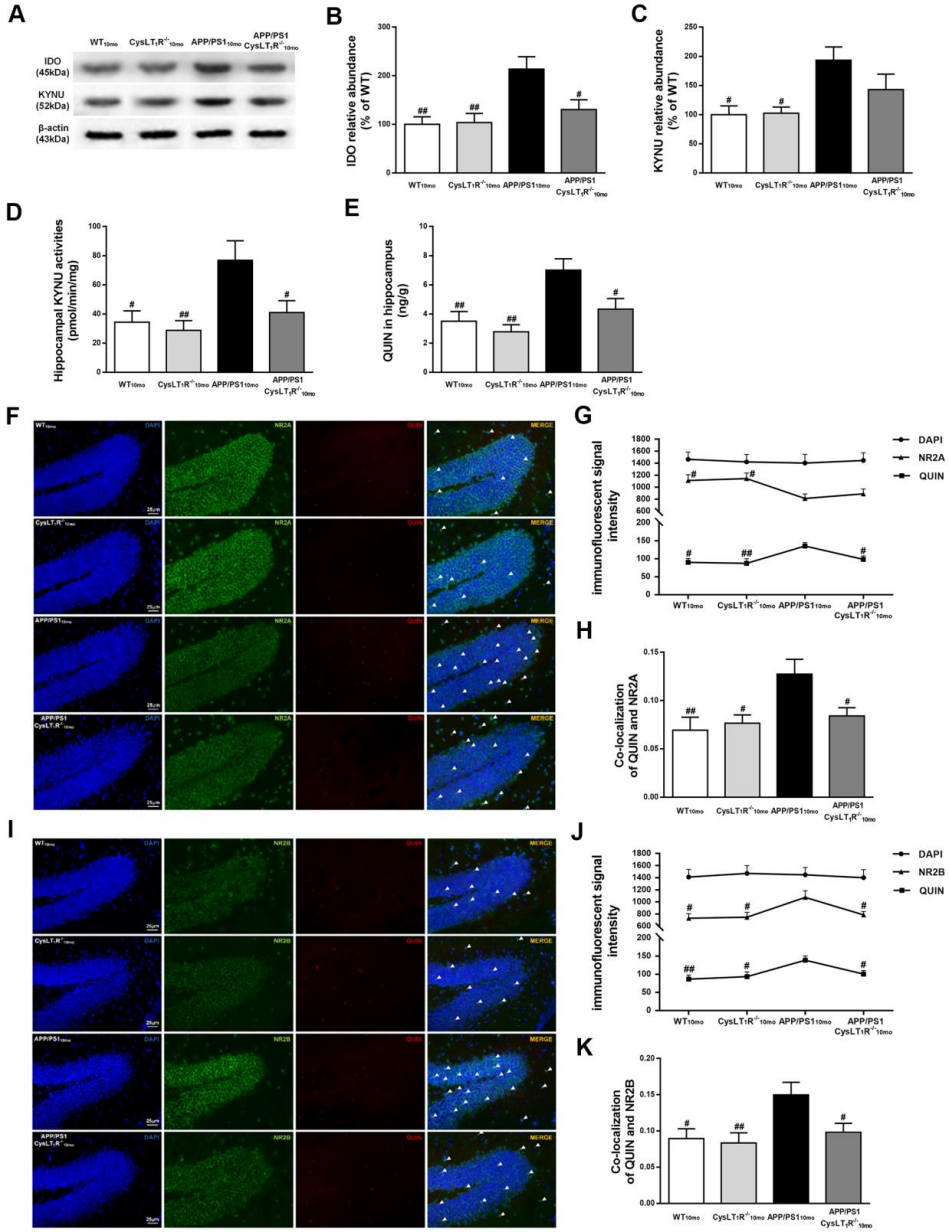
**Kynurenine pathway is involved in CysLT_1_R-mediated synaptic dysfunction.** (**A**) Representative immunoblots of IDO and KYNU protein in mice hippocampus. Quantifications of (**B**) IDO and (**C**) KYNU protein levels were expressed as the ratio (in %) of the WT mice. (**D**) Hippocampal KYNU activities were assessed by HPLC. (**E**) QUIN content in the hippocampus was detected by LC-MS/MS. The colocalization of QUIN with (**F**) NR2A or (**I**) NR2B was measured by immunofluorescence, respectively. (**G**) The immunofluorescent signal intensity of QUIN, NR2A, and DAPI in the DG. (**J**) The immunofluorescent signal intensity of QUIN, NR2B, and DAPI in the DG. Quantifications of colocalization of QUIN with (**H**) NR2A or (**K**) NR2B in hippocampal DG of brain sections were analyzed. All values are mean expressed as mean ± SEM, n = 4, ^#^*P<0.05*, ^##^*P<0.01*, ^###^*P*<0.001 vs. APP/PS1 mice.

We next sought to investigate whether deleting CysLT_1_R could block KP. Indoleamine 2,3-dioxygenase (IDO), controlling the initial rate-determining step of KP, was elevated in APP/PS1 mice hippocampus (F [3,12] = 6.67, *P*<0.01, Figure 6A, 6B), while CysLT_1_R deficiency reversed elevated expression of IDO in APP/PS1 mice (*P*<0.05). In additions, increased KYNU level was observed in APP/PS1 mice hippocampus (F [3,12] = 4.879, *P*<0.05, Figure 6A–6C), whereas CysLT_1_R deletion led to a slight but not significant decrease in KYNU level in APP/PS1 mice hippocampus (*P* > 0.05). Importantly, CysLT_1_R deletion markedly inhibited KYNU activity in APP/PS1 mice hippocampus (F [3,12] = 5.296, *P* < 0.05, Figure 6D).

QUIN content, determined by LC-MS/MS, increased to 7.01 ± 0.78 ng/g (F [3,12] = 7.499, *P* < 0.01, Figure 6E) in APP/PS1 mice hippocampus compared to 3.51 ± 0.66 ng/g in the WT mice, whereas it decreased to 4.33 ± 0.73 ng/g in APP/PS1-CysLT_1_R^-/-^ mice hippocampus (*P* < 0.05). Further, we performed double immunofluorescence staining to identify the colocalization of QUIN with NMDAR subunits in the DG (Figure 6F–6I). The immunofluorescent signal intensity of NR2A in the DG of APP/PS1 mice was markedly reduced (F [3, 44] = 3.602, *P* < 0.05, Figure 6G), whereas CysLT_1_R deletion didn’t affect the signal intensity of NR2A in the DG (*P* > 0.05). By contrast, the immunofluorescent signal intensity of QUIN and NR2B in the APP/PS1 group increased significantly (for QUIN, F [3, 44] = 4.708, *P* < 0.01; for NR2B, F [3, 44] = 3.822, *P* < 0.05, Figure 6J). CysLT_1_R deletion decreased the immunofluorescent signal intensity of QUIN (*P*<0.05) and NR2B (*P*<0.05). Furthermore, we found more frequent colocalization of QUIN with NR2A (F [3, 44] = 4.846, *P* < 0.01, Figure 6H) or NR2B (F [3, 44] = 4.434, *P* < 0.05, Figure 6K) in the DG of APP/PS1 mice hippocampus, respectively, whereas CysLT_1_R deletion inhibited the colocalization of QUIN with NR2A (*P* < 0.05) or NR2B (*P* < 0.05) in APP/PS1-CysLT_1_R^-/-^ mice. The above results demonstrate that KP may be involved in CysLT_1_R-mediated synaptic dysfunction.

### CysLT_1_R knockdown mediates similar effects on AD pathologies

To know whether CysLT_1_R knockdown could also yield similar effects seen in the knockout studies, we reduced CysLT_1_R levels in the DG of APP/PS1 mice hippocampus by bilateral injection of LV-CysLT_1_R shRNA-EGFP (Supplementary Figure 2A for protein, F [3, 12] = 9.678, *P* < 0.05, Supplementary Figure 2B, 2C; for mRNA, F [3, 12] = 13.92, *P* < 0.01, Supplementary Figure 2D, 2E). Moreover, immunohistochemical analyses also showed the CysLT_1_R levels was decreased in CysLT_1_R-shRNA-treated APP/PS1 mice hippocampus (F [3, 44] = 5.891, *P* < 0.05, Supplementary Figure 2F, 2G). In MWM test, escape latencies of training (day 1–5) is shown in Supplementary Figure 3A, 3B. On day 6, CysLT_1_R knockdown increased the percentage of time stayed in the target quadrant (F [3, 28] = 4.681, *P* < 0.05, Supplementary Figure 3C, 3E) and the numbers of platform crossings (F [3, 28] = 5.751, *P* < 0.05; Supplementary Figure 3D, 3E) in APP/PS1 mice. CysLT_1_R-shRNA-treated APP/PS1 mice also showed an increase in the number of correct choices (F [3, 28] = 4.201, *P* < 0.05, Supplementary Figure 3F) and a decrease in the escape latency to enter the safe compartment in Y-maze test (F [3, 28] = 5.189, *P* < 0.05, Supplementary Figure 3G). NORT suggested that CysLT_1_R-shRNA-treated APP/PS1 mice exhibited increased discrimination index (F [3, 28] = 5.954, *P* < 0.05; Supplementary Figure 3H). The mice in all groups traveled similar total distance in the open field (F[3,28]=0.131, P>0.05, Supplementary Figure 3I). Electrophysiological analysis demonstrated the improvement in LTP was associated with a concurrent restoration in dendritic spine density and synapses number in CysLT_1_R-shRNA-treated APP/PS1 mice (for LTP, F [3, 12] = 8.19, *P* < 0.05, Supplementary Figure 4A, 4B; for dendritic spine density, F [3, 16] = 5. 719, *P* < 0.05, Supplementary Figure 4C, 4D; for synapses number, F [3, 16] = 5.955, *P* < 0.05, Supplementary Figure 4E, 4F). In additions, hippocampal CysLT_1_R knockdown only increased the levels of PSD-95, but not of SYN, in APP/PS1 mice (for PSD-95, F [3, 12] = 6.969, *P* < 0.05, Supplementary Figure 4G, 4H; and for SYN, F [3, 12] = 5.506, *P* > 0.05, Supplementary Figure. 4G–4I). Moreover, Aβ_1-40_ and Aβ_1-42_ in both triton-soluble fraction and guanidine-HCl extraction from CysLT_1_R-shRNA-treated APP/PS1 mouse hippocampus were substantially reduced (triton-soluble: for Aβ_1-40_, F [1, 10] = 3.056, *P* < 0.05, and for Aβ_1-42_, F [1, 10] = 2.458, *P* < 0.05, Supplementary Figure 5A; guanidine-soluble: for Aβ_1-40_, F [1, 10] = 4.21, *P* < 0.05, and for Aβ_1-42_, F [1, 10] = 6.46, *P* < 0.05, Supplementary Figure 5B). CysLT_1_R knockdown markedly reduced Aβ deposition in APP/PS1 mice hippocampus (F [1, 22] = 3.079, *P* < 0.01, Supplementary Figure 5C, 5D). While CysLT_1_R knockdown did not alter hippocampal APP (F [3, 12] = 12.82, *P* > 0.05, Supplementary Figure 5E, 5F), levels of PS1 (F [3, 12] = 5.651, *P* < 0.05, Supplementary Figure 5E–5G), and BACE (F [3, 12] = 5.789, *P* < 0.05, Supplementary Figure 5E–5H) were decreased in the hippocampus of CysLT_1_R-shRNA-treated APP/PS1 mice. Moreover, hippocampal knockdown of CysLT_1_R had no effect on behavioral tests, hippocampal synaptic plasticity, and amyloidogenesis in WT mice.

## DISCUSSION

In our current study, elevated levels CysLT_1_R are firstly confirmed in the brains of AD patients and APP/PS1 mice. Increased levels of CysLT_1_R protein and mRNA were found in both hippocampus and prefrontal cortices, which were found to be age-dependent and region-specific with disease progression in APP/PS1 mice. Consistent with previous studies demonstrating age-dependent increases in glial and neuronal expressions of CysLT_1_R [17], our data also revealed APP/PS1 mice exhibited higher CysLT_1_R localization in microglia, astrocytes, and neurons compared with WT mice. How CysLT_1_R mediates AD-related deficits in APP/PS1 mice still remains elusive, though it is possible that accumulating Aβ induces the upregulation of 5-LOX [18], which in turn produces Cys-LTs, inducing CysLT_1_R expression.

Functional synapse formation is critical for the synaptic transmission in the brain, and synaptic plasticity is the important neurobiological foundation for learning and memory. Marked synapse loss was found in the brain, especially in hippocampus and cerebral cortex, of AD patients in previous findings. APP/PS1 mice at 3 months of age also showed deficits in synaptic plasticity [19]. In our study, we found impaired LTP, declined dendritic spine density, synapse number, and pre- and post-synaptic marker proteins in APP/PS1 mice. NMDAR, an ionotropic glutamate receptor in the hippocampus, is essential for cognition and dysfunction of NMDAR is closely correlated with excitotoxicity [15, 20]. In additions, NMDARs functions are influenced by interaction with PSD-95, an important postsynaptic scaffolding protein that is involved in protein assembly, synaptic development and neural plasticity [21]. The functions of NMDARs and PSD-95 play the critical role in LTP induction at CA3-CA1 synapses [22]. As such, NMDARs dysfunction and PSD-95 reduction in APP/PS1 mice may explain the corresponding LTP reduction we observed in the current study. We speculate that increased NR2B occurs in extrasynaptic locations in APP/PS1 mice, and overactivation of extrasynaptic NMDARs lead to a variety of aberrant transcriptional cascades and posttranslational modifications associated with neurotoxicity by stimulating cell death pathways [23]. Notably, we demonstrated that CysLT_1_R gene knockout or knockdown effectively restored LTP and reversed elevated NR2B levels as well as reduced PSD-95 expression in APP/PS1 mice hippocampus. Interestingly, despite the improvement in spine density, synapse number, and the levels of NMDARs due to CysLT_1_R deletion in APP/PS1 mice, CysLT_1_R^-/-^ mice did not exhibit any alterations compared to WT mice (Figure 2C–2L), suggesting CysLT_1_R may mediate neurotoxicity only under pathological conditions.

Aβ deposits develop in APP/PS1 mice from 6 months and it progressively increase with age. Abundant plaques are observed in both hippocampus and cortex of 9-month-old APP/PS1 mice and continue to increase to around 12 months of age [24]. In the present study, Aβ pathology was ameliorated through CysLT_1_R deficiency without altering total APP levels in APP/PS1 mice. Thus, we investigated whether APP processing and Aβ degradation were changed. Our data indicated that CysLT_1_R gene knockout or knockdown didn’t affect the level of CTFα produced by α-secretase cleavage in APP/PS1 mice, whereas CTFβ produced by BACE1 cleavage was decreased, which could be explained by a reduction in BACE1 levels. Additionally, PS1, the essential part of the γ-secretase complex, was reduced after CysLT_1_R gene knockout or knockdown, which might also explain the decrease in Aβ levels. Furthermore, the proteolytic enzymes, IDE and NEP, that degrade Aβ were not altered significantly by CysLT_1_R gene knockout or knockdown [25]. Moreover, we observed that APP/PS1-CysLT_1_R^-/-^ mice showed inhibited activation of astrocytes, microglia and decreased release of IL-6 and TNF-α. It is possible that the levels of BACE1 and PS1 are increased in reactive astrocytes of AD brain. Our previous studies have suggested that CysLT_1_R promotes the release of proinflammatory cytokines though NF-κB signaling pathway, and proinflammatory cytokines upregulate BACE1 activity and Aβ production [26]. Therefore, CysLT_1_R deletion ameliorates amyloidogenesis in APP/PS1 mice most likely by decreasing the proinflammatory cytokines expression and inhibiting astrocytes activation via NF-κB signaling. Moreover, recent study has indicated that Aβ clearance is promoted by converting microglia from the M1 state to the M2 state [27]. The activation states of microglia could be M1 phenotype representing pro-inflammatory activity or M2 microglia exhibiting an anti-inflammatory phenotype. Therefore, the markers for M1 phenotype could be pro-inflammatory cytokines, such as TNFα and IL-1β, and the markers for M2 phenotype could be transforming growth factor-β (TGF-β) against inflammation [28]. Importantly, the marker of M1 microglia phenotype, TNFα, was elevated in APP/PS1 mice but decreased by CysLT_1_R deletion, implicating a possible shift of microglia phenotype from M1 to M2. Consequently, CysLT_1_R deficiency could promote Aβ clearance by microglial phagocytosis.

Besides NF-κB signaling pathway and following neuroinflammation and apoptosis mediated by CysLT_1_R, our data revealed that KP induced by proinflammatory cytokines is also involved in the CysLT_1_R-mediated AD pathogenesis. Substantial evidence has accumulated in the past decades depicting that dysregulation of KP and the production of neurotoxic metabolites are major contributors to the pathogenesis of several neurodegenerative diseases such as AD [29]. The initial rate-limiting step of the KP from tryptophan is determined by Indoleamine 2,3-dioxygenase (IDO) activity. The proinflammatory cytokines, such as IFNγ, TNF-α and IL-1β, synergistically stimulate IDO expression and activity [30–32]. Beyond this, RelB-p52 could directly bind to the IDO promoter region and subsequently induce IDO transcription in noncanonical NF- kB pathway [33]. Aβ induces IDO expression and increased levels of IDO have been observed in hippocampal tissue from AD patients. Consistent with these studies, our data showed IDO level was elevated in APP/PS1 mice at 10 months age. As a key enzyme lying on the main pathway towards NAD^+^ synthesis, KYNU oxidizes both L-KYN and 3-OH-L-KYN to anthranilic acid and 3-OH-ANA, respectively [34], followed by the synthesis of QUIN. QUIN, a specific competitive agonist for NMDAR [35], is identified as a potential neurotoxin causing glutamatergic excitotoxicity [36]. Thus, upregulation of KYUN would favor the production of increased amount of toxic QUIN. As expected, we found elevated KYNU levels and activities in APP/PS1 mice, and such increases were reversed through CysLT_1_R knockout. Importantly, QUIN impairs postsynaptic elements, promotes neurodegenerative lesions, as well as induces neuroinflammation and apoptosis, which are involved in the complex and multifactorial cascade leading to neurodegeneration [37]. Previous studies revealed that CysLT_1_R antagonist montelukast significantly attenuated striatal lesion, oxidative stress and mitochondrial dysfunction in rats caused by intrastriatal administration of QUIN [38]. Therefore, we have suggested QUIN is likely a major contributor to the impairment of synaptic plasticity and cognitive deficits in AD and blockade of CysLT_1_R might have neuroprotective potential. Our data indicated significant increases in QUIN content and more intensive colocalization with NR2A or NR2B in APP/PS1 mice, and the content and colocalization were attenuated by CysLT_1_R deletion. These data may explain why CysLT_1_R deficiency enhances hippocampal synaptic plasticity in APP/PS1 mice.

**Figure 7:**
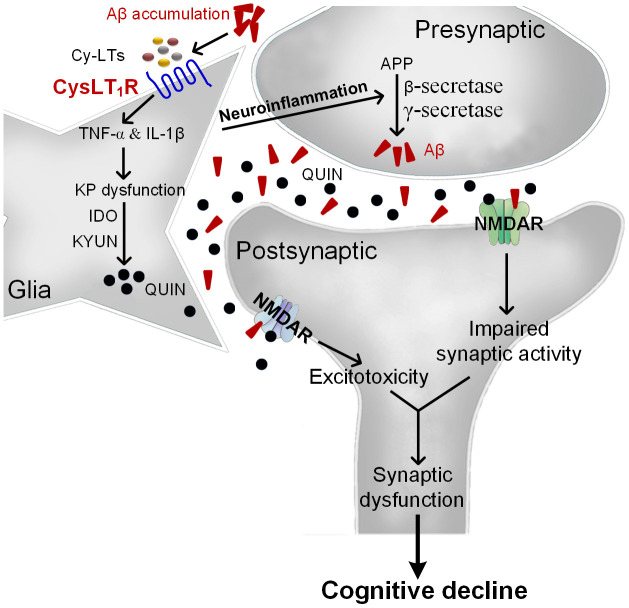
**The proposed mechanism for CysLT_1_R-mediated AD pathology.** Aβ accumulation upregulates Cys-LTs inducing CysLT_1_R expression, which activates NF-κB pathway followed by increased release of proinflammatory cytokines. Consequently, proinflammatory cytokines induce neuronal apoptosis and KP dysregulation with increased expression of IDO and KYUN and the synthesis of QUIN. Moreover, neuroinflammation accelerates amyloid deposition, forming a vicious circle. This leads to NMDARs overactivation and excitotoxicity correlated with synaptic dysfunction and cognitive deficits.

Overall, we propose that Aβ accumulation upregulates Cys-LTs inducing CysLT_1_R expression, which activates NF-κB pathway followed by increased release of proinflammatory cytokines. Consequently, proinflammatory cytokines induce neuronal apoptosis and KP dysregulation with increased IDO and KYUN and the synthesis of QUIN. Moreover, neuroinflammation accelerates amyloid deposition, forming a vicious circle. These lead to NMDARs overactivation and excitotoxicity correlated with synaptic dysfunction and cognitive deficits (Figure 7). However, CysLT_1_R gene knockout ameliorates amyloidogenesis and cognitive impairment through inhibiting neuroinflammation and neurotoxic effects of QUIN on NMDARs. More importantly, CysLT_1_R gene knockdown using LV-shRNA in APP/PS1 mice confirms the results obtained from APP/PS1-CysLT_1_R^-/-^ mice. LV-CysLT_1_R shRNA generates stable knockdown in the hippocampus, which is regional specificity and selectivity. CysLT_1_R knockdown mediated by LV-CysLT_1_R shRNA also overcomes the possible compensation and avoids gene deletion-caused adverse effect on physiological function and lifetime. These experiments have been performed in a combination of CysLT_1_R knockdown and knockout, and the data suggest that downregulation of CysLT_1_R might be an effective therapeutic strategy for AD. The present study expends our understanding about the mechanism underlying CysLT_1_R-mediated AD pathology and suggests CysLT_1_R as a potential target for AD therapy.

## MATERIALS AND METHODS

### Materials

A list of the antibodies used in this experiment is listed in the supplementary materials. Pyfidoxal-5'-phosphate, 3-hydroxyanthranilic acid and 3-hydroxykynurenine were purchased from Sigma-Aldrich Co. LLC.

### Animals

Male APP/PS1ΔE9 mice (herein referred to as APP/PS1) and their wild-type (WT) littermates, CysLT_1_R^-/-^ mice and APP/PS1-CysLT_1_R^-/-^ mice were constructed and bred by Model Animal Research Center of Nanjing University (Nanjing, China). The constitutive CysLT_1_R knockout (CysLT_1_R^-/-^) mice were generated with the aid of CRISPR/Cas9-mediated gene editing in mice of C57BL/6 background. CysLT_1_R^-/-^ mice developed normally and were fertile while they showed abnormal vascular permeability. CysLT_1_R^-/-^ mice were further bred with APP/PS1 mice to generate APP/PS1-CysLT_1_R^-/-^ mice. RT-PCR was used for genotyping and the following primers were used: CysLT_1_R forward 5’-GAATGGAACTGAAAATCTGACGAC-3’ and reverse 5’-ATAATAGACCACACGGAGAGGCA-3’, APP forward 5’-GACTGACCACTCGACCAGGTTCTG-3’ and reverse 5’-CTTGTAAGTTGGATTCTCATATCCG-3’, forward 5’-AATAGAGAACGGCAGGAGCA-3’ and reverse 5’-GCCATGAGGGCACTAATCAT-3’. Identifications for CysLT_1_R^-/-^ mice and APP/PS1-CysLT_1_R^-/-^ mice have been provided in Supplementary Material II and III, respectively. The animals were kept at 22° C and a 12 h light-dark cycle with unrestricted food and water supplies. Care of animals and handling were done according to the National Institutes of Health Guide for the Care and Use of Laboratory Animals (NIH Publications, revised, 2011), and with approval from the Animal Care and Use Committee of China Pharmaceutical University. Group allocation during the animal experiments were blinded to the investigators for unbiased reporting of the outcomes.

### Human AD brain samples

Post-mortem brain sections were dissected from frozen brains of AD patients (about 80-87 years old, Chinese male patients) and age-matched controls (about 78-87 years old, Chinese male patients), which were provided by South Central University for Nationalities (Wuhan, China). Informed consents were obtained from the subjects’ family members. Pathological and clinical criteria were matched for the diagnosis of AD. The post-mortem interval of the brain tissue collection was between 2 and 4 h. The use of brain tissue samples was approved by the ethics committee of China Pharmaceutical University and was in accordance with the ethical standards of the institutional and national research committee and with the 1964 Helsinki declaration.

### Lentivirus generation

Lentiviral shRNA was used to silence CysLT1R gene according to a previous study [35]. The lentiviral vector construct expressing the short hairpin RNA (shRNA) was generated which was complementary to the coding exon of the mice CysLT1R gene and tagged with a fused enhanced green fluorescent protein (EGFP). The construct was labelled as LV-CysLT1R shRNA. A lentiviral vector expressing EGFP alone (LV-EGFP) was used as the control. The sequence for the CysLT1R miRNA (shRNA-mir hairpin structure) was 5′-TGCATCAAATTGTTGCTTTTTCAAGAGAAAAGCAACAATTTGATGCAttttttc-3′ (the antisense target sequence in bold, while the sense target sequence in italics). The normal control (NC) sequence from Genechem was 5′-TTCTCCGAACGTGTCACGTTTCAAGAGAACGTGACACGTTCGGAGAAtttttg-3′. The underlined 9 nt in the middle represents the hairpin loop in both the CysLT1R shRNA and NC sequences. PCR was used to amplify the coding sequence of the CysLT1R shRNA. The primer sequences were: 5′-GCCCCGGTTAATTTGCATAT-3′ (forward) and 5′-GAGGCCAGATCTTGGGTG-3′ (reverse). All of the lentiviral vectors contained EGFP as a reporter to track lentivirus-mediated expression.

### Stereotaxic injection of lentivirus in mouse brain

Both WT and APP/PS1 mice at 9 months of age were anaesthetized with 350 mg/kg chloral hydrate. Bilateral hippocampal dentate gyrus (DG) injection of LV-CysLT_1_R shRNA-EGFP was performed stereotactically at coordinates 2.1 mm to posterior, 1.7 mm to lateral, and 2.1 mm to ventral relative to brain bregma of APP/PS1 mice. LV-EGFP served as the control vector. The viral suspension at a volume of 2.5 μl (2.5×10^6^ vector genome) was injected into each injection site using a 10 μl glass syringe at a rate of 250 nl/min. The needle was left untouched for 10 min and then removed slowly over 2 minutes to avoid backflow of the suspension. The mice were kept warm until the surgical recovery. After 4 weeks, the mice underwent behavioral testing and biochemical examinations.

### Western blot

Western blot was performed as described previously [10]. RIPA buffer was used to homogenize the isolated hippocampi and its formulation details have been listed in Supplementary Table 2. The total protein concentration was determined by a BCA assay kit from Beyotime (China). The details of antibodies have been listed in Supplementary Table 3.

### RT-PCR assay

Total RNA was extracted according to the manufacturer's protocol. The mouse hippocampi were homogenized using Trizol reagents. Formulation details of reaction system has been listed in Supplementary Table 4. This reaction mixture was then incubated for 5 min at 25° C, for 60 min 42° C, and then at for 10 min 72° C to deactivate the reverse transcriptase. PCR mixture was prepared with cDNA template (10 μl) dissolved into 40 μl reaction mixture containing 10 × PCR buffer (4.0 μl), 25 mM MgCl_2_ (3.0 μl), primer (12.5 pmol), and Taq DNA polymerase (0.25 μl). The cycling parameters (Supplementary Tables 5, 6) and primer sequences (Supplementary Table 7) have been listed in supplementary materials. The amplified products were separated on an ethidium bromide-containing agarose gel (1.5 %) by electrophoresis and then photographed. The bands were checked by an image analysis system from Tanon Science and Technology Co. Ltd. and the PCR was performed on an Eppendorf Master Cycler from Eppendorf (Germany).

### Immunohistochemistry and immunofluorescence

Following transcardial perfusion mice brains were fixed with 4 % paraformaldehyde at 4° C for 2 days, and then equilibrated in 30 % sucrose at 4° C for 1 day and 30 μm sections were made. For immunohistochemistry, the sections were washed with PBS (0.1 M, 3 × 5 min) and then treated with H_2_O_2_ (3 %, 10 min). They were washed again in PBS (0.1 M, 3 × 5 min) and treated with Triton X-100 (0.3 %, 30 min). After washing again with PBS (0.1 M, 3 × 5 min), the sections were blocked with the solution containing 0.3 % Triton X-100 and 5 % BSA for 1 h. Then the sections were incubated with primary antibodies (details of the antibodies listed in Supplementary Table 8) overnight at 4° C. Next morning, sections were incubated with biotinylated anti-rabbit lgG or anti-mouse lgG at 37° C for 20 min, and again after washing with PBS, incubated with streptavidin-biotin complex at 37° C for 20 min. Diaminobenzidine was used to detect target proteins. Finally, the sections were gradient dehydrated and photographed and images were quantified using Image J and Image-Pro Plus software. The integrated optical density of CysLT_1_R expression or Aβ deposition (% area occupied) was calculated based on quantification. The mean values of 3 sections from each animal were analyzed.

For immunofluorescence, after an overnight blocking in 2 % serum and 0.3 % triton, sections were incubated for 48 h or 72 h in primary antibody (details of the antibodies listed in Supplementary Table 8). The primary antibodies were localized with corresponding affinity-purified IgG. The sections were examined using a fluorescence microscope from Leica. Three sections were randomly analyzed to calculate the % colocalization.

### ELISA

Aβ detection by ELISA was performed as described previously [39]. Fractions of the hippocampus (100-120 mg) were homogenized in 5 × mass of ice-cold lysis buffer containing Tris-HCl (10 mM), EDTA (5 mM) and proteinase inhibitor cocktail in 320 mM sucrose (pH 7.4). Then the homogenate was lysed on ice for 15 min and then centrifugated at 4° C (14,000 g, 15 min), leaving the supernatants containing triton-soluble Aβ peptides. The pellets which remained insoluble were re-homogenized using 10 volumes of 5 M guanidine-HCl (diluted in 50 mM tris-HCl, pH 8.0) and shaken for 4 h at room temperature. Homogenates were centrifuged (8,000 g, 5 min). The triton-soluble and guanidine-HCl-soluble fractions were used for final detection of Aβ_40_ or Aβ_42_ according to the manufacturer’s manual. For IL-6, and TNF-α detection, 100 mg hippocampal tissue was rinsed with PBS and homogenized in 1 ml PBS followed by overnight storage at -20° C. Cell membranes of the homogenate were broken through two freeze-thaw cycles and the homogenates were centrifuged at 2-8° C (5,000 g, 5 min). The supernatants were collected and assayed immediately. The ELISA kits for Aβ_1-40_ and Aβ_1-42_ were from Cusabio Biotech Co. Ltd. (China), and for IL-6 and TNF-α from Neobioscience Co. Ltd. (China).

### Hippocampal slice preparation and electrophysiology

Transversal hippocampal slices from WT and APP/PS1 mice at 10 months of age were prepared and placed in a recording chamber with continuous ACSF perfusion at a rate of 3 ml/min with at 24° C. Cells were visualized with an upright microscope and Schaffer collaterals was stimulated by a tungsten monopolar electrode. The field excitatory postsynaptic potentials (fEPSPs) were recorded under current-clamp mode from the CA1 stratum radiatum by a glass microelectrode filled with ACSF (3–4 MΩ). A high-frequency stimulation protocol consisting of two one-second long 100 Hz trains was used to induce long term potentiation (LTP) [40].

### Golgi staining

FD Rapid Golgi Stain Kit (FD Neuro Technologies, USA) was used for Golgi staining [41]. Coronal brain blocks were made from unfixed brain samples and were immersed in a solution containing solution A and B with the volume ratio of 1 to 1 at room temperature for 2 weeks followed by soaking in solution C at 4° C for 48 h. Then the brain samples were frozen with dry-ice powder and serially sectioned into 100 μm coronal slices (containing the hippocampus) with a freezing microtome [42, 43]. These frozen sections were mounted with solution C on a 0.5 % gelatin-coated glass slide and allowed to dry naturally at room temperature. Then they were immersed in the solutions containing solution D, solution E, and distilled water at a ratio of 1:1:2 for 5 min, followed by washing with distilled water twice for 4 min each. The sections were cover slipped after dehydrating with graded alcohol solutions and clearing with xylene. The preparations were observed under a microscope. For morphological analysis of hippocampal DG neurons, 5 granule neurons from each mouse (4 mice/group, 20 neurons from each group) were calculated from the hippocampal DG. Only neurons with non-truncated dendrites, and consistent and dark staining along the dendrites were selected for analysis; they were also selected only if there was clear isolation from neighboring neurons in order to avoiding interference with analysis [44, 45]. Dendritic spine densities were estimated on secondary dendritic branches of each imaged neuron in clearly evaluable areas, approximately 150-200 μm from the soma. Only branches over 20 μm in length were included in the study. The number of spines was quantified by ImageJ. Spine density was calculated per 10 μm of dendritic length.

### Electron microscopy

Synaptic density was detected by electron microscopy [42]. Following transcardial perfusion with PBS containing 2 % glutaraldehyde and 3 % paraformaldehyde (under anesthesia), fixed brains were taken out and hippocampal slices were then prepared. The slices were fixed again with cold OsO_4_ (1 %, 1h). Ultrathin sections (90 nm) were made and stained with uranyl acetate and lead acetate, and viewed at 100 kV on a JEOL 200CX electron microscope. Synapses were identified by the presence of synaptic vesicles and postsynaptic densities.

### HPLC for hippocampal KYNU activity

Isolated hippocampi were sonicated in 9 × mass of 0.1 M PBS and centrifuged (12,000 g, 30 min) at 4° C. Protein in supernatants was determined by BCA protein assay and was stored at -80° C until use. For total 3-hydroxyanthranilic acid (3-HANA) content assay, 1 volume of pyfidoxal-5'-phosphate (PLP) solution (48 μM PLP in barbitone sodium-hydrochloric acid buffer, pH 8.4) was added into 5 volumes of hippocampal lysates, and 6 volumes of 3-hydroxykynurenine (3-HK, 2 mM), a substrate of KYNU, were immediately added into the mixture and incubated for 8 min at 30° C. 12 volumes of trichloroacetic acid were added to terminate the reaction. The mixture was thoroughly mixed and centrifuged (13,200 g, 20 min) at 40° C. The levels of 3-HANA, the product of 3-HK, in the supernatants were determined by HPLC. For basal 3-HANA content assay, 12 volumes of trichloroacetic acid were added into 5 volumes of hippocampal lysates. The mixture was thoroughly mixed and centrifuged (13,200 g, 20 min) at 40° C and then the supernatants were collected and determined by HPLC. The actual content of 3-HANA was calculated by subtracting the basal 3-HANA content from the total 3-HANA content. KYNU activity (pmol/min/mg) was expressed as actual content of 3-HANA divided by reaction time and total protein concentrations in the reaction system.

Chromatographic separation was carried out on C18 column (4.6 mm × 250 mm, I.D., 5 μm). The mobile phase was 0.1 M KH_2_PO_4_ buffer containing l % acetonitrile. The column temperature was set at 30° C with a flow rate of 1.0 mL/min, and the injection volume is 50 μl. The fluorescent intensity was detected at 322 nm (excitation wavelength) and 414 nm (emission wavelength).

### LC-MS/MS for QUIN content

Liquid chromatography coupled to tandem mass spectrometry (LC-MS/MS) was performed to measurement of QUIN and 2,4-Pyridinedicarboxylic acid was used as internal standard. Hippocampi were sonicated in 9 × mass of 0.2 % acetic acid in aqueous solution containing internal standard and centrifuged (12,000 g, 15 min) at 4° C. After centrifugation (15000 g, 60 min) at 4° C, the supernatants were then filtered through a 3 kDa Amico Ultra filter. The LC-MS/MS system consisted of an Acquity UPLC system (Waters Corp, Inc., CA, USA) and a Waters Xevo TQ-S triple quadrupole mass spectrometer equipped with an electrospray ionization source interface operated in the positive multiple reaction monitoring mode. Ion source settings were: capillary voltage, 3.00 kV; desolation temperature, 450° C; source temperature, 150° C; nitrogen was used as the desolation gas with an API gas flow rate of 1000 L/h. Quantification was operated in MRM of transition m/z 168→78, and MassLynx V4.1 software (Waters Corp, Inc., CA, USA) was used to data acquisition and processing. Chromatography was performed on a Zorbax Eclipse XDB-C8 column (100 mm × 4.6 mm, i.d. 3.5 μm). Gradient methods with a total duration of 6 min each were used for chromatographic separation (Mobile A: 0.5 % formic acid in aqueous; Mobile B: 1 % formic acid in acetonitrile). The flow rate for detection of both analytes was 0.3 mL/min. Retention time for the analytes was 1.17 min. Average concentrations were based on quadruplicate measures. All samples and regents were of HPLC grade.

### Behavioral tests

Spatial learning and memory were assessed by Morris water maze (MWM) [46], and working memory was assessed by Y-maze [47] and novel object recognition test (NORT) [48]. Open field test (OFT) was used for assessing the general locomotor activity [49]. These behavioral tests were performed and analyzed as described previously.

### Statistical analyses

Repeated measure ANOVA was used to analyze group differences in MWM escape latency with “days” as the within-subject factor and “group” as the between-subject factor. Other data were analyzed using either Student’s t-test (for two-group comparison) or one-way ANOVA (for comparison among more than two groups) followed by a Dunnett’s post-hoc analysis, if necessary. Mean ± standard error of the mean (SEM) was used for representation of the descriptive data. All analyses were carried out using SPSS, version 20.0. Statistical significance was considered when *P*-value was < 0.05.

## Supplementary Material

Supplementary Figures

Supplementary Tables

Supplementary Material II

Supplementary Material III
