# Supplementary material for: CRISPR/Cas9-mediated CysLT1R deletion reverses synaptic failure, amyloidosis and cognitive impairment in APP/PS1 mice
Source: Aging (Albany NY). 2021 Feb 11;13(5):6634–61. doi: 10.18632/aging.202501 (PMC7993729; doi:10.18632/aging.202501)
Supplement: Supplementary Figures [file aging-13-202501-s001.pdf]

SUPPLEMENTARY FIGURES

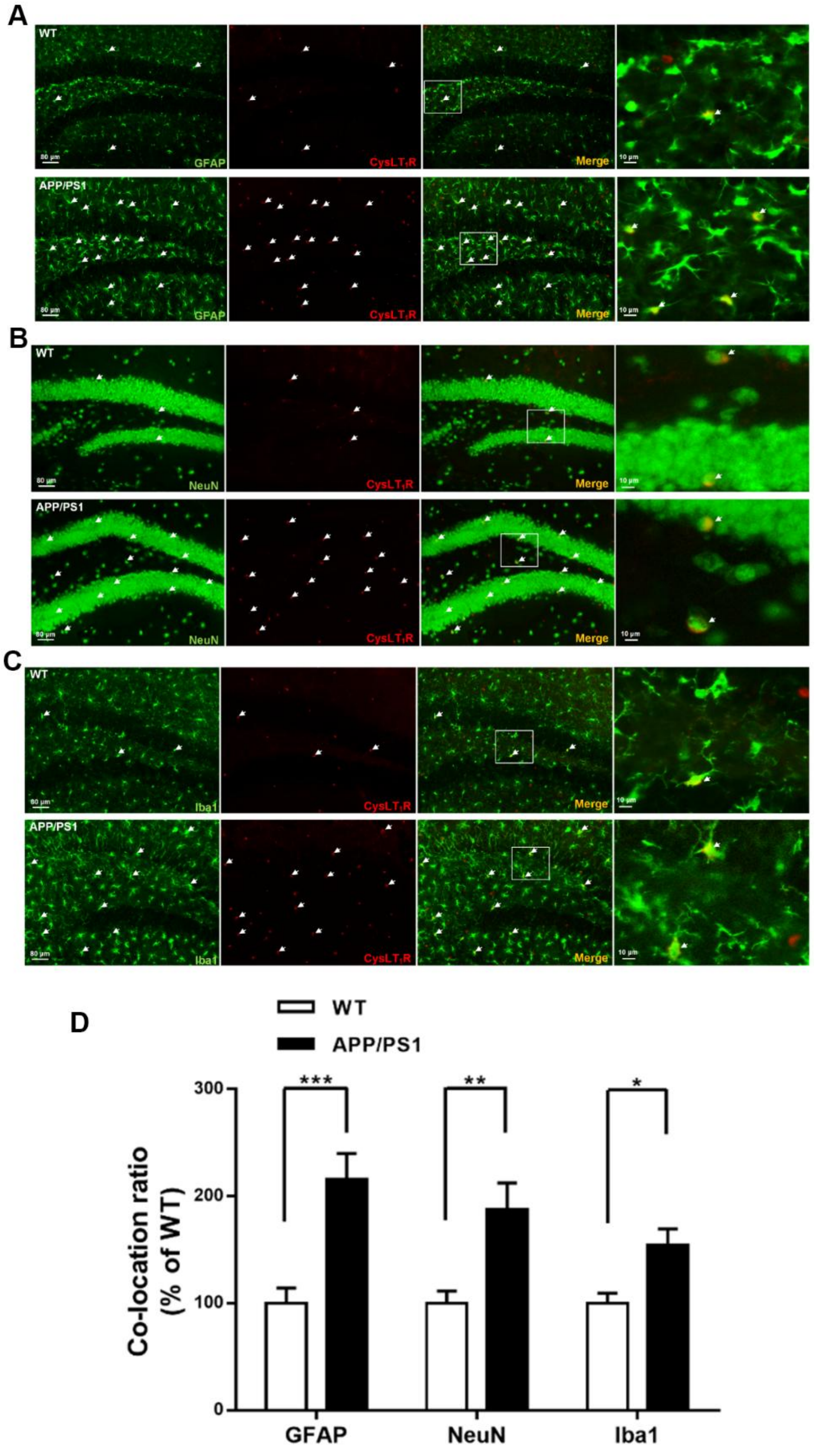

Supplementary Figure 1. CysLT$_1$R expression is upregulated in different cells of the brains from APP/PS1 mice. (**A**–**C**) CysLT$_1$R expression in neuron, astrocyte and microglia of the hippocampal DG in APP/PS1 mice and littermate control at the age of 10 months. Scale bar, 50 µm. (**D**) Quantification of CysLT$_1$R in the brain sections of mice. Values are mean ± SEM, n = 4, **P<0.05*, ***P<0.01*, ***P<0.001* vs. WT mice.

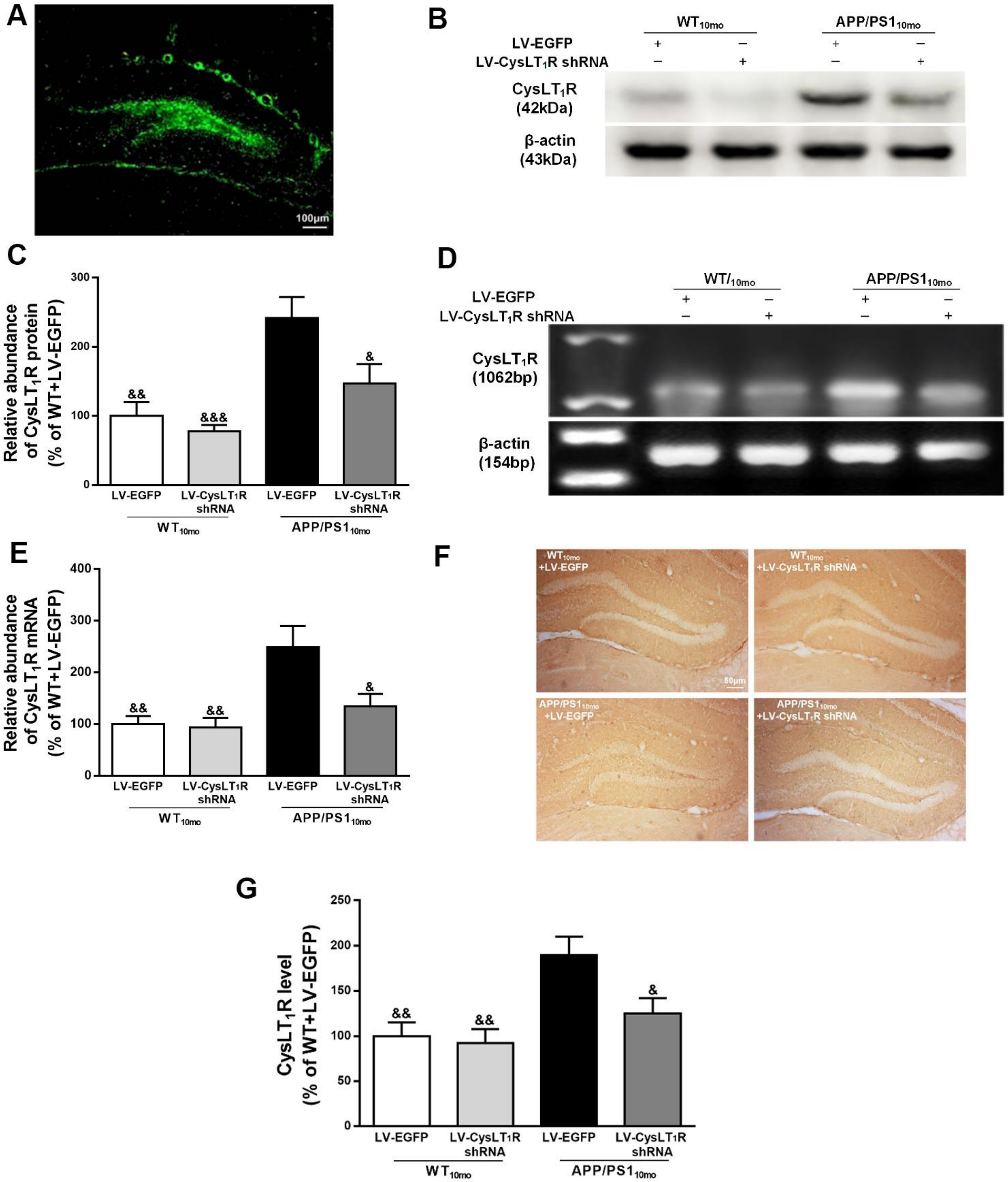

Supplementary Figure 2. Hippocampal CysLT$_1$R knockdown by injection with the LV-CysLT$_1$R shRNA-EGFP. (**A**) Shown is representative hippocampal DG with lentivirus transfection after 4 weeks. (**B**) WB detection of CysLT$_1$R protein in the hippocampi of 10-month-old APP/PS1 mice injected with the LV-CysLT$_1$R shRNA-EGFP or LV-EGFP bilaterally into the DG. (**C**) Quantification of CysLT$_1$R protein level was expressed as the ratio (in %) of WT+LV-EGFP group. (**D**) RT-PCR detection of CysLT$_1$R mRNA in the hippocampi of WT and APP/PS1 mice injected with the LV-CysLT$_1$R shRNA-EGFP or LV-EGFP bilaterally into the DG. (**E**) Quantification of CysLT$_1$R mRNA level was expressed as the ratio (in %) of WT+LV-EGFP group. (**F**) Immunohistochemical analyses of CysLT$_1$R levels in the hippocampi of 10-month-old APP/PS1 mice injected with the LV-CysLT$_1$R shRNA-EGFP or LV-EGFP bilaterally into the DG. Scale bar = 50 µm. (**G**) Quantification of CysLT$_1$R in the brain sections of mice. All values are expressed as mean ± SEM, n = 4, $^{\&}$*P<0.05*, $^{\&\&}$*P<0.01*, $^{\&\&}$*P<0.001* vs. APP/PS1+LV-EGFP mice.

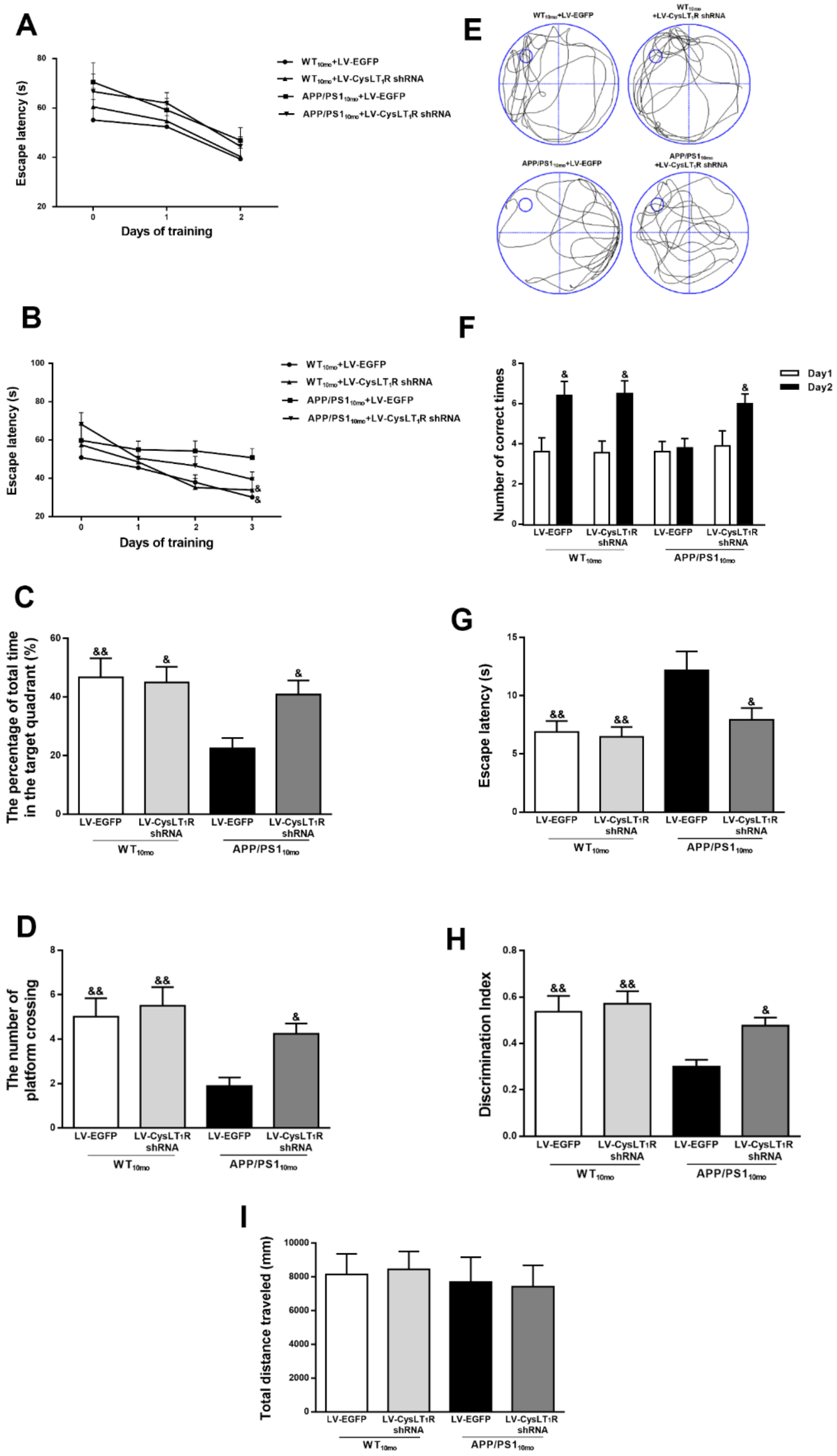

Supplementary Figure 3. Hippocampal knockdown of CysLT$_1$R improves cognitive decline in APP/PS1 mice. In the MWM task, day 0 indicates performance in the first trial, and subsequent points represent average of all daily trials. (**A**) The mean escape latency to the visible platform (**B**) The mean escape latency to the hidden platform (**C**) The percentage of time spent in the target quadrant, and (**D**) numbers of platform location crossings during the probe trial test. (**E**) Representative swim paths of mice. In the Y-maze test, (**F**) the number of correct choices on days 1-2 and (**G**) the latency to enter the shock-free compartment on day 2. In NORT, (**H**) discrimination index shown by the time spent exploring the novel object compared to the familiar one. In open field test, (**I**) the total distance traveled was analyzed. All values are expressed as mean ± SEM, n = 8, $^{\&}$*P<0.05*, $^{\&\&}$*P<0.01*, $^{\&\&}$*P<0.001* vs. APP/PS1+LV-EGFP mice.

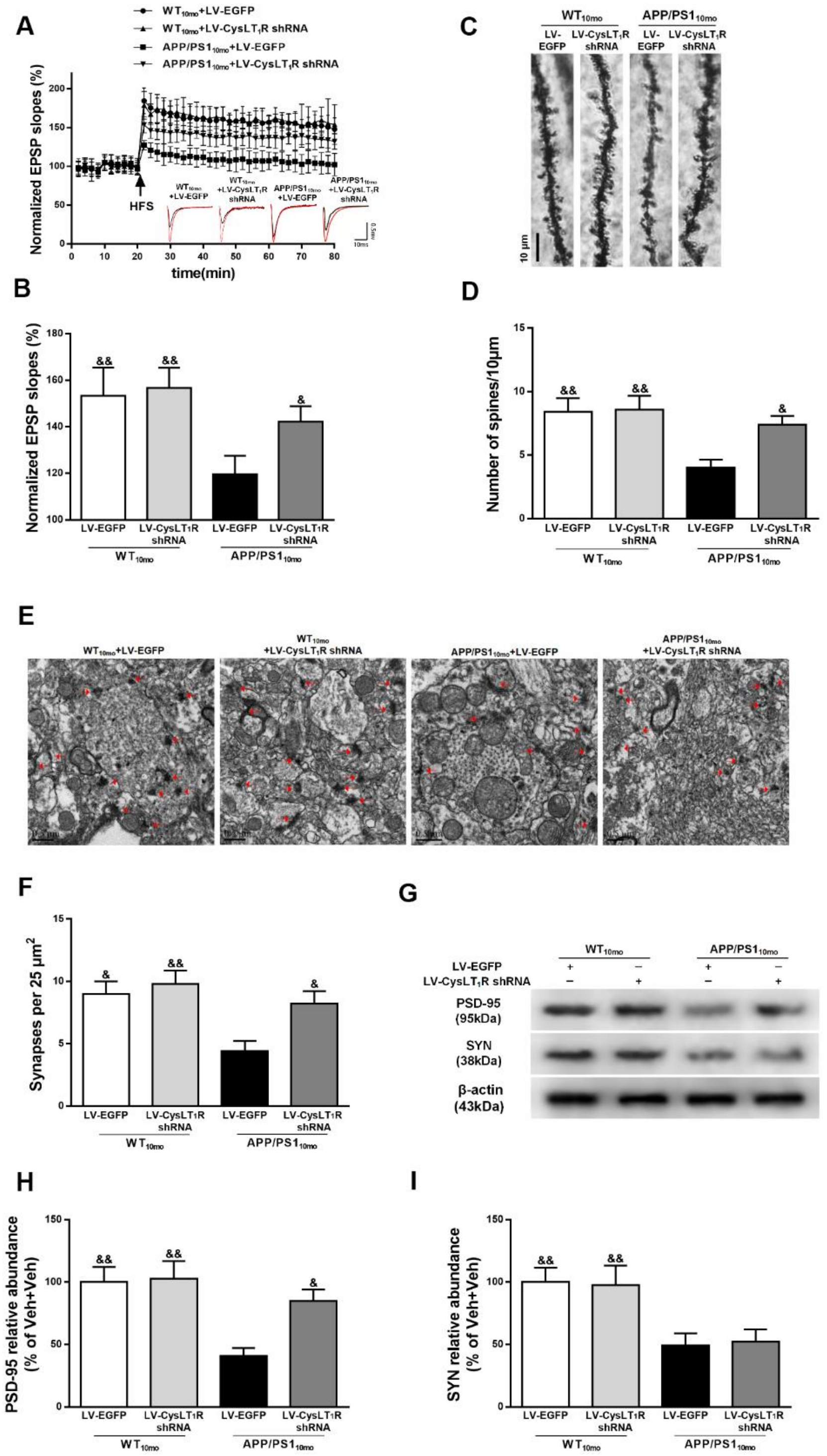

Supplementary Figure 4. Hippocampal knockdown of CysLT$_1$R improves hippocampal synaptic plasticity in APP/PS1 mice. (**A**) The induction of hippocampal LTP was assessed after high-frequency stimulation (HFS; indicated as an arrow) and recorded for 60 min post-induction. (**B**) Summary bar-graphs showing differences in mean values of fEPSPs slope during 55-60 mins following the induction of LTP among genotypes. (**C**) Representative images of Golgi-impregnated dendrites in the hippocampi of 10-month-old APP/PS1 mice injected with the LV- CysLT$_1$R shRNA-EGFP or LV-EGFP bilaterally into the DG. Scale bar = 10 μm. (**D**) Statistical analysis of the average number of dendritic spines. (**E**) The synaptic density in the hippocampus of WT and APP/PS1 mice injected with the LV- CysLT$_1$R shRNA-EGFP or LV-EGFP bilaterally into the DG. Scale bar = 1 μm. (**F**) Statistical analysis of synaptic density calculated as the number of synapses per 25 μm^2. (**G**) Representative immunoblots of PSD-95 and SYN in the hippocampi of 10-month-old APP/PS1 mice injected with the LV- CysLT$_1$R shRNA-EGFP or LV-EGFP bilaterally into the DG. Quantification of (**H**) PSD-95 and (**I**) SYN protein levels were expressed as the ratio (in %) of WT+LV-EGFP group. All values are expressed as mean ± SEM, n = 4-5, $^{\&}P<0.05$, $^{\&\&}P<0.01$, $^{\&\&}P<0.001$ vs. APP/PS1+LV-EGFP mice.

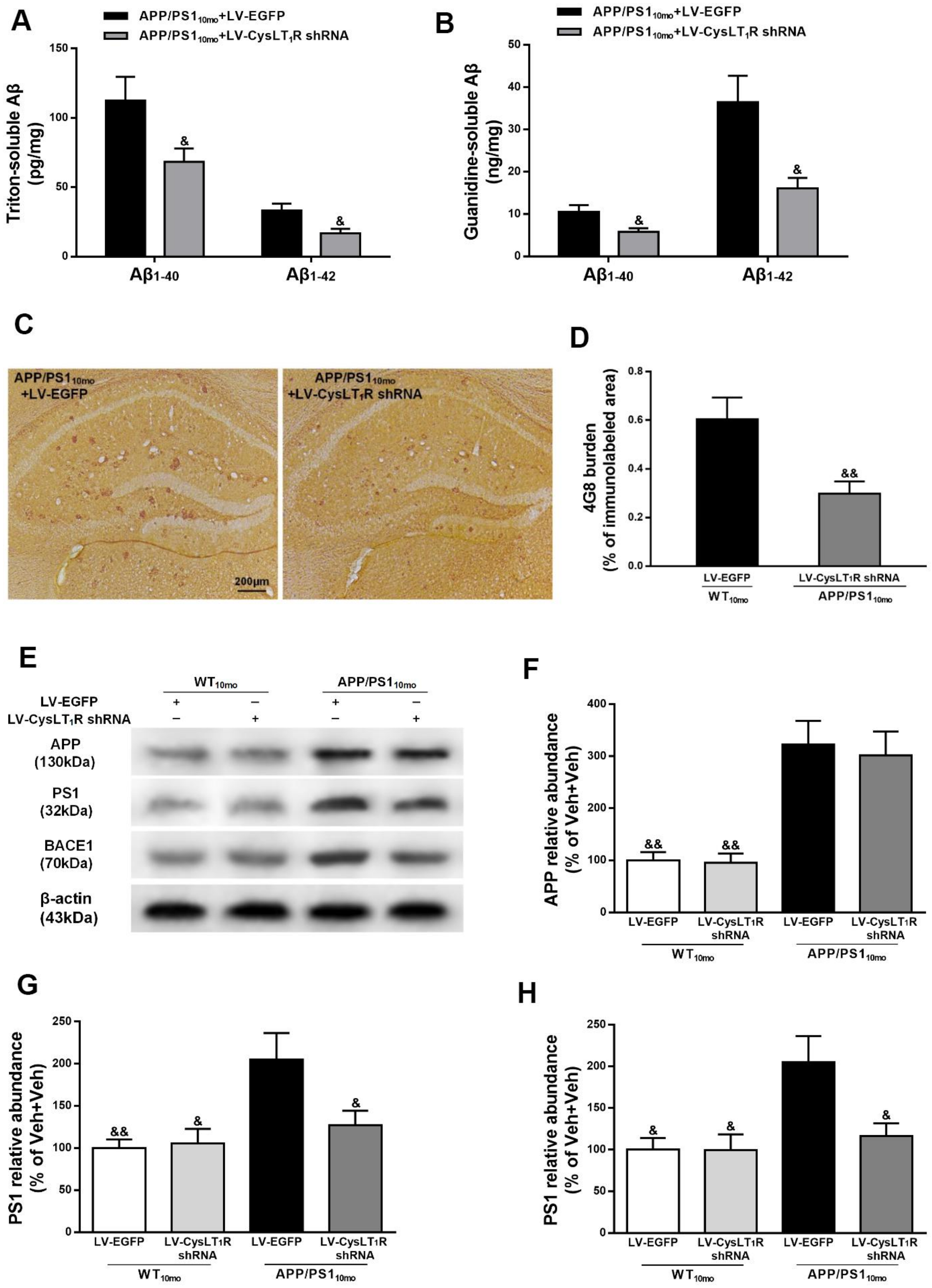

Supplementary Figure 5. Hippocampal knockdown of CysLT$_1$R inhibits amyloidogenesis in APP/PS1 mice. The triton-soluble fractions (**A**) and the guanidine-soluble fractions (**B**) of $A\beta^{1-}{}_{40}$ and $A\beta^{1-}{}_{42}$ in the hippocampi of 10-month-old APP/PS1 mice injected with the LV-CysLT$_1$R shRNA-EGFP or LV-EGFP bilaterally into the DG were assessed by ELISA. (**C**) Aβ immunostaining with 4G8 antibody in brain sections. Scale bar = 200 μm. (**D**) The percentage of area covered by Aβ deposition was quantified. (**E**) Representative immunoblots of APP, PS1 and BACE in the hippocampi of WT and APP/PS1 mice injected with the LV- CysLT$_1$R shRNA-EGFP or LV-EGFP bilaterally into the DG. Quantifications of (**F**) APP, (**G**) PS1 and (**H**) BACE were expressed as the ratio (in %) of WT+LV-EGFP group. Values are mean ± SEM, n = 4-6, $^{\&}$*P<0.05*, $^{\&\&}$*P<0.01*, $^{\&\&}$*P<0.001* vs. APP/PS1+LV-EGFP mice.
