# Supplementary material for: CRISPR/Cas9-mediated CysLT1R deletion reverses synaptic failure, amyloidosis and cognitive impairment in APP/PS1 mice
Source: Aging (Albany NY). 2021 Feb 11;13(5):6634–61. doi: 10.18632/aging.202501 (PMC7993729; doi:10.18632/aging.202501)
Supplement: Supplementary Tables [file aging-13-202501-s002.pdf]

SUPPLEMENTARY TABLES

Supplementary Table 1. The detail information of AD patients and age-matched controls.

No.	Age	Sex	Bw (g)	Pmi (h)	ApoE	Braak	area	DNC
1	83	male	1007	4:00	43	5	prefrontal cortex	AD, dehydration
2	84	male	1021	3:20	33	5	prefrontal cortex	AD, cachexia and dehydration
3	80	male	1195	3:00	33	6	prefrontal cortex	AD, pneumonia
4	87	male	1010	2:55	43	5	prefrontal cortex	AD, heart failure
5	80	male	1257	3:30	33	1	prefrontal cortex	AG, lung carcinomas
6	87	male	1315	4:00	33	2	prefrontal cortex	AG, heart failure
7	81	male	1311	3:15	32	0	prefrontal cortex	AG, glioblastoma multiforme, coma
8	78	male	1330	2:30	43	2	prefrontal cortex	AG, pulmonary emphysema

Bw = brain weight, Pmi = post-mortem interval, DNC = diagnosis, neuropathology and cause of death.

Supplementary Table 2. RIPA buffer used for Western blot.

Components of RIPA buffer	Concentration
Tris–HCl (pH 7.4)	50 mM
NaCl	150 nM
PMSF	1 mM
EDTA	1 mM
Triton X-100	1 %
sodium deoxycholate	1 %
SDS	0.1 %

Supplementary Table 3. Antibodies used for Western blot.

Antigen	Antibody type	Source	Titer
CysLT1R	mouse	Santa Cruz Biotechnology, Inc.	1:500
APP	rabbit	Cell Signaling Technology, Inc	1:1000
BACE	rabbit	Cell Signaling Technology, Inc	1:1000
PS1	rabbit	Cell Signaling Technology, Inc	1:1000
PSD-95	rabbit	Cell Signaling Technology, Inc	1:1000
SYN	rabbit	Millipore Inc	1:1000
KYNU	rabbit	R&D system Co., Ltd	1:500
NR2A	mouse	BD transduction laboratories Co.	1:1000
NR2B	mouse	BD transduction laboratories Co.	1:1000
IDO	rabbit	Abcam Co., Ltd	1:1000
β-actin	rabbit	Boster Biotechnology Co., Ltd	1:5000

Supplementary Table 4. Reaction system used for PCR.

Reaction Components	Volume (μL)
random hexamer primer contained 5 μg of total RNA	1.0
5 × reaction buffer	4.0
dNTP Mixture (10 mM)	2.0
ribonuclease inhibitor	0.5
200 UM-MuLV reverse transcriptase in reverse reaction buffer	2.0
Milli-Q H_2O	To 20μL

Supplementary Table 5. CysLT$_1$R cycling parameters used for PCR.

Temperature	Time	Cycle
94° C	1 min	
94° C	30 s	
63° C	30 s	33
72° C	1 min	
72° C	7 min	
25° C	hold	

Supplementary Table 6. β-actin cycling parameters used for PCR.

Temperature	Time	Cycle
94° C	1 min	
94° C	30 s	
55° C	30 s	25
72° C	1 min	
72° C	7 min	
25° C	hold	

Supplementary Table 7. Primer sequences for RT-PCR.

Target gene	Forward primer (5'-3')	Reverse primer (5'-3')
CysLT$_1$R	ATTCCTGGAGAACATGAATGG	CATTGTTCTGCACTGTAGATGAG
β-actin	TCTTGGGTATGGAATCCTGTG	ATCTCCTTCTGCATCCTGTCA

For CysLT$_1$R, 1062 bp, nucleotides 419-1480 in NM_021476.4, GeneBank.
For β-actin, 154 bp, nucleotides 876–1029 in NM_007393.3, GeneBank.

Supplementary Table 8. Antibodies used for immunohistochemistry and immunofluorescence.

Antigen	Antibody type	Source	Titer
CysLT1R	rabbit	Cayman Chemical	1:100
4G8	mouse	Covance, Inc	1:100
NeuN	rabbit	Cell Signaling Technology, Inc	1:100
GFAP	rabbit	Sigma-Aldrich Co., LLC	1:100
CD68	rabbit	Abcam Co., Ltd	1:500
NR2A	mouse	BD transduction laboratories Co.	1:100
NR2B	mouse	BD transduction laboratories Co.	1:100
QUIN	rabbit	Abcam Co., Ltd	1:500
primary antibody	Alexa Fluor 488 donkey anti-rabbit IgG	Boster Biotechnology Co., Ltd	1:200
primary antibody	Alexa Fluor 594 goat anti-mouse IgG	Jackson Laboratory	1:200
