# Supplementary material for: CRISPR/Cas9-mediated CysLT1R deletion reverses synaptic failure, amyloidosis and cognitive impairment in APP/PS1 mice
Source: Aging (Albany NY). 2021 Feb 11;13(5):6634–61. doi: 10.18632/aging.202501 (PMC7993729; doi:10.18632/aging.202501)

Supplementary Material II. Identification of Cysltr1-/- mice.

**Identifications for Cysltr1^-/-^ mice**

**Notes**

Cysltr1 with X-chromosomal inheritance Wt=253bp -50bp=203bp

**Primers**

for Cysltr1 identification

2322-Cysltr1-F1-tF1 GAATGGAACTGAAAATCTGACGAC

2322-Cysltr1-F1-tR1 ATAATAGACCACACGGAGAGGCA

**PCR reaction system**

| **Reaction Components** | **Volume (μL)** |
| --- | --- |
| gDNA template | 2.0 |
| 10×*Taq* buffer (mg^2+^ plus) | 2.0 |
| dNTP mixture (10 mM) | 0.5 |
| Primer mixture (10 μM) | 0.5 |
| *Taq* DNA polymerase (5 U/µL) | 0.5 |
| Milli-Q H_2_O | To 20μL |

PCR programs

| **Temperature** | **Time** | **Cycle** |
| --- | --- | --- |
| 95^o^C | 5 min |  |
| 95 ^o^C | 30 s | 20 |
| 65^o^C | 30 s |
| 72 ^o^C | 30 s |
| 95 ^o^C | 30 s | 20 |
| 55 ^o^C | 30 s |
| 72 ^o^C | 30 s |
| 72 ^o^C | 3 min |  |
| 25 ^o^C | hold |  |

**Gel concentration**

3.0%

**Electrophoretic pattern**
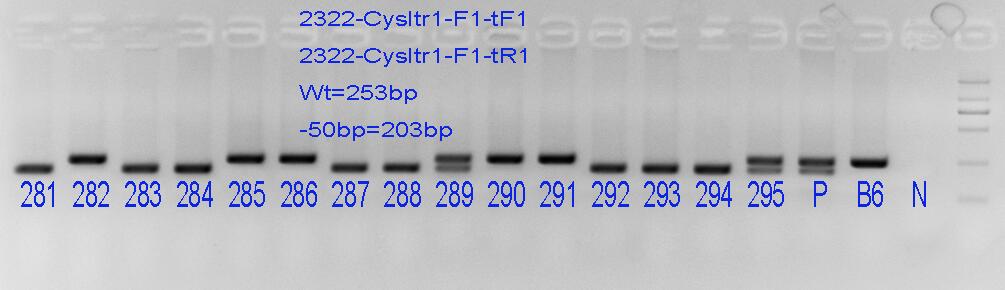


**Result adjudication**

-50bp/-50bp or -50bp/Y: 281, 283, 284, 287, 288, 292-294

-50bp/wt: 289, 295

Wt/Y: the rest (for male)


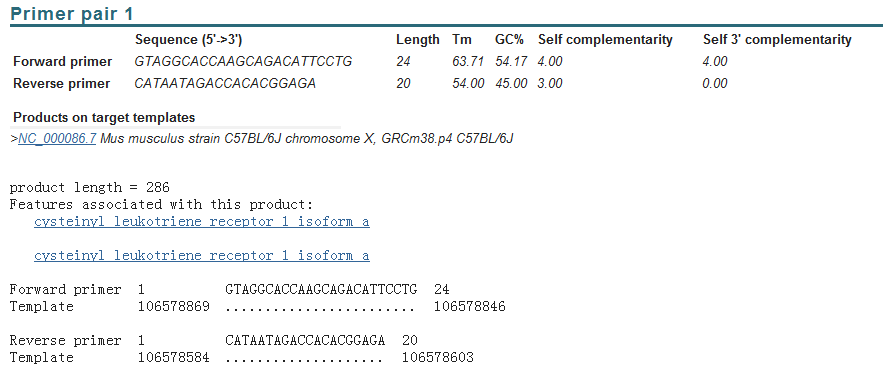


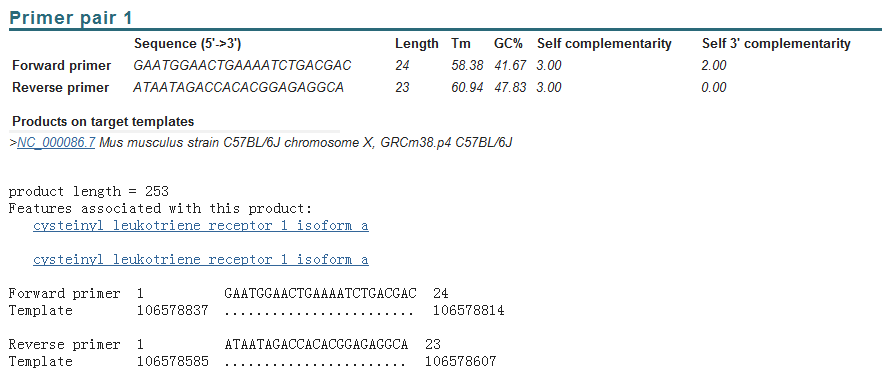

Supplement: Supplementary Material II [file aging-13-202501-s003.doc]
