# Supplementary material for: CRISPR/Cas9-mediated CysLT1R deletion reverses synaptic failure, amyloidosis and cognitive impairment in APP/PS1 mice
Source: Aging (Albany NY). 2021 Feb 11;13(5):6634–61. doi: 10.18632/aging.202501 (PMC7993729; doi:10.18632/aging.202501)
Supplement: Supplementary Material III [file aging-13-202501-s004.doc]

Supplementary Material III. Identification of APP/PS1 Cysltr1-/- mice.

**Identifications for APP/PS1 Cysltr1^-/-^ mice**

**Primers**

for Cysltr1 identification

Wt=253bp -50bp=203bp

2322-Cysltr1-F1-tF1 GAATGGAACTGAAAATCTGACGAC

2322-Cysltr1-F1-tR1 ATAATAGACCACACGGAGAGGCA

for APP identification

TG=350bp WT=None

oIMR1597 GACTGACCACTCGACCAGGTTCTG

oIMR1598 CTTGTAAGTTGGATTCTCATATCCG

for PSEN1 identification

TG=608bp WT=None

oIMR1644 AATAGAGAACGGCAGGAGCA

oIMR1645 GCCATGAGGGCACTAATCAT

PCR reaction system

| **Reaction Components** | **Volume （μL）** | |
| --- | --- | --- |
| gDNA template | 2.0 |  |
| 10×*Taq* buffer (mg^2+^ plus) | 2.0 |  |
| dNTP mixture (10 mM) | 0.5 |  |
| Primer mixture (10 μM) | 0.5 |  |
| *Taq* DNA polymerase (5 U/µL) | 0.5 |  |
| Milli-Q H_2_O | To 20μL |  |

**PCR programs**

P1

| **Temperature** | **Time** | **Cycle** |
| --- | --- | --- |
| 95^o^C | 5 min |  |
| 95 ^o^C | 30 s | 20 |
| 65^o^C | 30 s |
| 72 ^o^C | 30 s |
| 95 ^o^C | 30 s | 20 |
| 55 ^o^C | 30 s |
| 72 ^o^C | 30 s |
| 72 ^o^C | 3 min |  |
| 25 ^o^C | hold |  |

P2, P3

| **Temperature** | | **Time** | | **Cycle** |
| --- | --- | --- | --- | --- |
| 95^o^C | 5 min | |  | |
| 95 ^o^C | 30 s | | 40 | |
| 60^o^C | 30 s | |
| 72 ^o^C | 30 s | |
| 72 ^o^C | 3 min | |  | |
| 25 ^o^C | hold | |  | |

**Gel concentration**

3.0% for P1, 1.5% for P2 and P3

**Electrophoretic pattern**

**
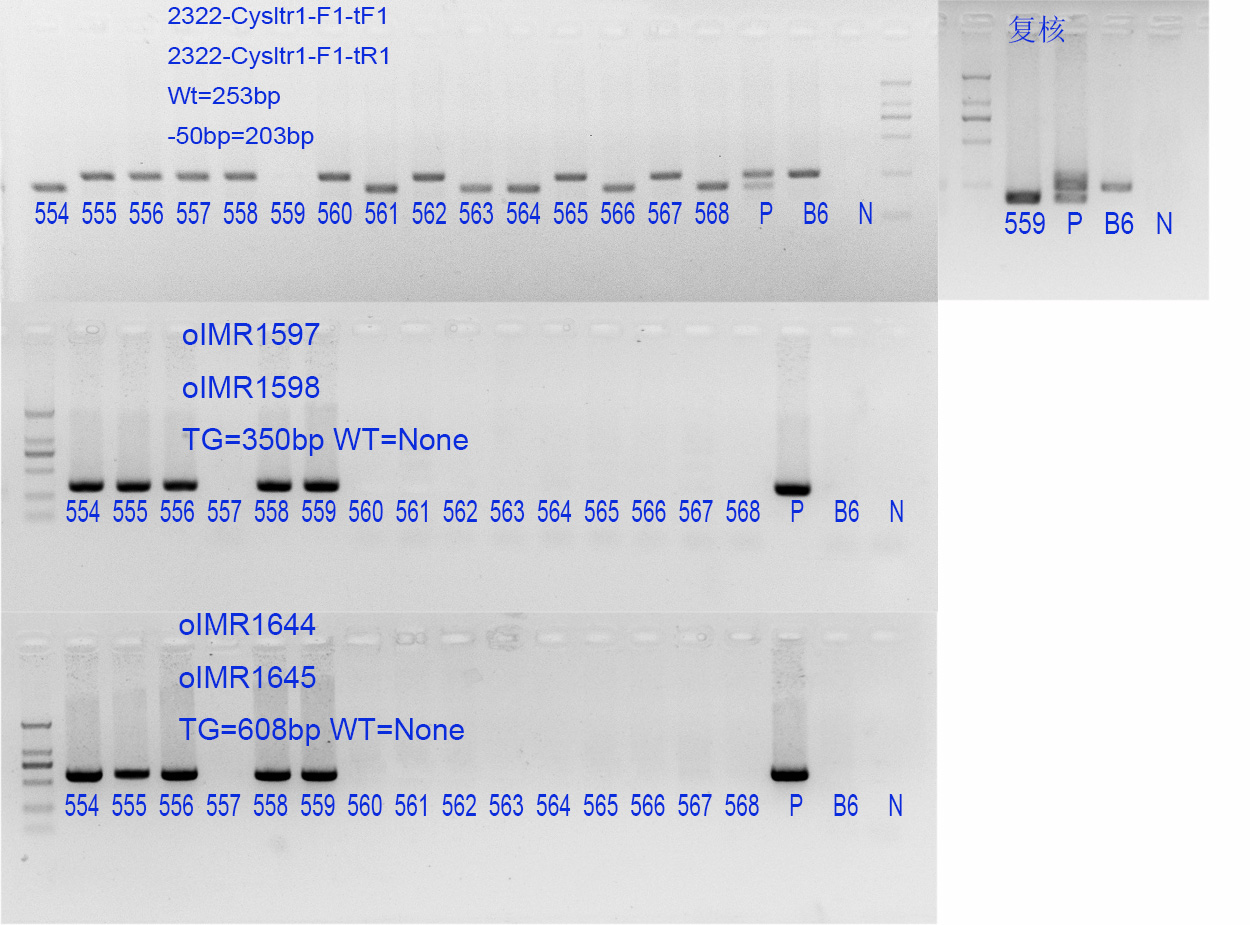
**

**Result adjudication**

identification for Cysltr1

-50bp/-50bp or-50bp/Y: 554, 559, 561, 563, 564, 566, 568

WT/WT or WT/Y: the rest (for male)

identification for APP and PSEN1

TG: 554-556, 558, 559

WT: the rest
